# Community assessment to advance computational prediction of cancer drug combinations in a pharmacogenomic screen

**DOI:** 10.1038/s41467-019-09799-2

**Published:** 2019-06-17

**Authors:** Michael P. Menden, Dennis Wang, Mike J. Mason, Bence Szalai, Krishna C. Bulusu, Yuanfang Guan, Thomas Yu, Jaewoo Kang, Minji Jeon, Russ Wolfinger, Tin Nguyen, Mikhail Zaslavskiy, AstraZeneca-Sanger Drug Combination DREAM Consortium, Jordi Abante, Barbara Schmitz Abecassis, Nanne Aben, Delasa Aghamirzaie, Tero Aittokallio, Farida S. Akhtari, Bissan Al-lazikani, Tanvir Alam, Amin Allam, Chad Allen, Mariana Pelicano de Almeida, Doaa Altarawy, Vinicius Alves, Alicia Amadoz, Benedict Anchang, Albert A. Antolin, Jeremy R. Ash, Victoria Romeo Aznar, Wail Ba-alawi, Moeen Bagheri, Vladimir Bajic, Gordon Ball, Pedro J. Ballester, Delora Baptista, Christopher Bare, Mathilde Bateson, Andreas Bender, Denis Bertrand, Bhagya Wijayawardena, Keith A. Boroevich, Evert Bosdriesz, Salim Bougouffa, Gergana Bounova, Thomas Brouwer, Barbara Bryant, Manuel Calaza, Alberto Calderone, Stefano Calza, Stephen Capuzzi, Jose Carbonell-Caballero, Daniel Carlin, Hannah Carter, Luisa Castagnoli, Remzi Celebi, Gianni Cesareni, Hyeokyoon Chang, Guocai Chen, Haoran Chen, Huiyuan Chen, Lijun Cheng, Ariel Chernomoretz, Davide Chicco, Kwang-Hyun Cho, Sunghwan Cho, Daeseon Choi, Jaejoon Choi, Kwanghun Choi, Minsoo Choi, Martine De Cock, Elizabeth Coker, Isidro Cortes-Ciriano, Miklós Cserzö, Cankut Cubuk, Christina Curtis, Dries Van Daele, Cuong C. Dang, Tjeerd Dijkstra, Joaquin Dopazo, Sorin Draghici, Anastasios Drosou, Michel Dumontier, Friederike Ehrhart, Fatma-Elzahraa Eid, Mahmoud ElHefnawi, Haitham Elmarakeby, Bo van Engelen, Hatice Billur Engin, Iwan de Esch, Chris Evelo, Andre O. Falcao, Sherif Farag, Carlos Fernandez-Lozano, Kathleen Fisch, Asmund Flobak, Chiara Fornari, Amir B. K. Foroushani, Donatien Chedom Fotso, Denis Fourches, Stephen Friend, Arnoldo Frigessi, Feng Gao, Xiaoting Gao, Jeffrey M. Gerold, Pierre Gestraud, Samik Ghosh, Jussi Gillberg, Antonia Godoy-Lorite, Lizzy Godynyuk, Adam Godzik, Anna Goldenberg, David Gomez-Cabrero, Mehmet Gonen, Chris de Graaf, Harry Gray, Maxim Grechkin, Roger Guimera, Emre Guney, Benjamin Haibe-Kains, Younghyun Han, Takeshi Hase, Di He, Liye He, Lenwood S. Heath, Kristoffer H. Hellton, Manuela Helmer-Citterich, Marta R. Hidalgo, Daniel Hidru, Steven M. Hill, Sepp Hochreiter, Seungpyo Hong, Eivind Hovig, Ya-Chih Hsueh, Zhiyuan Hu, Justin K Huang, R. Stephanie Huang, László Hunyady, Jinseub Hwang, Tae Hyun Hwang, Woochang Hwang, Yongdeuk Hwang, Olexandr Isayev, Oliver Bear Don’t Walk, John Jack, Samad Jahandideh, Jiadong Ji, Yousang Jo, Piotr J. Kamola, Georgi K. Kanev, Loukia Karacosta, Mostafa Karimi, Samuel Kaski, Marat Kazanov, Abdullah M Khamis, Suleiman Ali Khan, Narsis A. Kiani, Allen Kim, Jinhan Kim, Juntae Kim, Kiseong Kim, Kyung Kim, Sunkyu Kim, Yongsoo Kim, Yunseong Kim, Paul D. W. Kirk, Hiroaki Kitano, Gunter Klambauer, David Knowles, Melissa Ko, Alvaro Kohn-Luque, Albert J. Kooistra, Melaine A. Kuenemann, Martin Kuiper, Christoph Kurz, Mijin Kwon, Twan van Laarhoven, Astrid Laegreid, Simone Lederer, Heewon Lee, Jeon Lee, Yun Woo Lee, Eemeli Lepp_aho, Richard Lewis, Jing Li, Lang Li, James Liley, Weng Khong Lim, Chieh Lin, Yiyi Liu, Yosvany Lopez, Joshua Low, Artem Lysenko, Daniel Machado, Neel Madhukar, Dries De Maeyer, Ana Belen Malpartida, Hiroshi Mamitsuka, Francesco Marabita, Kathleen Marchal, Pekka Marttinen, Daniel Mason, Alireza Mazaheri, Arfa Mehmood, Ali Mehreen, Magali Michaut, Ryan A. Miller, Costas Mitsopoulos, Dezso Modos, Marijke Van Moerbeke, Keagan Moo, Alison Motsinger-Reif, Rajiv Movva, Sebastian Muraru, Eugene Muratov, Mushthofa Mushthofa, Niranjan Nagarajan, Sigve Nakken, Aritro Nath, Pierre Neuvial, Richard Newton, Zheng Ning, Carlos De Niz, Baldo Oliva, Catharina Olsen, Antonio Palmeri, Bhawan Panesar, Stavros Papadopoulos, Jaesub Park, Seonyeong Park, Sungjoon Park, Yudi Pawitan, Daniele Peluso, Sriram Pendyala, Jian Peng, Livia Perfetto, Stefano Pirro, Sylvia Plevritis, Regina Politi, Hoifung Poon, Eduard Porta, Isak Prellner, Kristina Preuer, Miguel Angel Pujana, Ricardo Ramnarine, John E. Reid, Fabien Reyal, Sylvia Richardson, Camir Ricketts, Linda Rieswijk, Miguel Rocha, Carmen Rodriguez-Gonzalvez, Kyle Roell, Daniel Rotroff, Julian R. de Ruiter, Ploy Rukawa, Benjamin Sadacca, Zhaleh Safikhani, Fita Safitri, Marta Sales-Pardo, Sebastian Sauer, Moritz Schlichting, Jose A. Seoane, Jordi Serra, Ming-Mei Shang, Alok Sharma, Hari Sharma, Yang Shen, Motoki Shiga, Moonshik Shin, Ziv Shkedy, Kevin Shopsowitz, Sam Sinai, Dylan Skola, Petr Smirnov, Izel Fourie Soerensen, Peter Soerensen, Je-Hoon Song, Sang Ok Song, Othman Soufan, Andreas Spitzmueller, Boris Steipe, Chayaporn Suphavilai, Sergio Pulido Tamayo, David Tamborero, Jing Tang, Zia-ur-Rehman Tanoli, Marc Tarres-Deulofeu, Jesper Tegner, Liv Thommesen, Seyed Ali Madani Tonekaboni, Hong Tran, Ewoud De Troyer, Amy Truong, Tatsuhiko Tsunoda, Gábor Turu, Guang-Yo Tzeng, Lieven Verbeke, Santiago Videla, Daniel Vis, Andrey Voronkov, Konstantinos Votis, Ashley Wang, Hong-Qiang Horace Wang, Po-Wei Wang, Sheng Wang, Wei Wang, Xiaochen Wang, Xin Wang, Krister Wennerberg, Lorenz Wernisch, Lodewyk Wessels, Gerard J. P. van Westen, Bart A. Westerman, Simon Richard White, Egon Willighagen, Tom Wurdinger, Lei Xie, Shuilian Xie, Hua Xu, Bhagwan Yadav, Christopher Yau, Huwate Yeerna, Jia Wei Yin, Michael Yu, MinHwan Yu, So Jeong Yun, Alexey Zakharov, Alexandros Zamichos, Massimiliano Zanin, Li Zeng, Hector Zenil, Frederick Zhang, Pengyue Zhang, Wei Zhang, Hongyu Zhao, Lan Zhao, Wenjin Zheng, Azedine Zoufir, Manuela Zucknick, In Sock Jang, Zara Ghazoui, Mehmet Eren Ahsen, Robert Vogel, Elias Chaibub Neto, Thea Norman, Eric K. Y. Tang, Mathew J. Garnett, Giovanni Y. Di Veroli, Stephen Fawell, Gustavo Stolovitzky, Justin Guinney, Jonathan R. Dry, Julio Saez-Rodriguez

**Affiliations:** 1 Oncology, IMED Biotech Unit, AstraZeneca, Cambridge, SG8 6EH UK; 2 European Bioinformatics Institute, European Molecular Biology Laboratory, Cambridge, CB10 1SD UK; 3 Sheffield Institute for Translational Neuroscience, University of Sheffield, Sheffield, S10 2TN UK; 4 Sage Bionetworks, Seattle, WA 98121 USA; 5 Department of Physiology, Faculty of Medicine, Semmelweis University, Budapest, 1085 Hungary; 6 Laboratory of Molecular Physiology, Hungarian Academy of Sciences and Semmelweis University (MTA-SE), Budapest, 1085 Hungary; 7 RWTH Aachen University, Faculty of Medicine, Joint Research Center for Computational Biomedicine, Aachen, 52062 Germany; 8 Department of Computational Medicine and Bioinformatics, University of Michigan, Ann Arbor, 48109 USA; 9 Department of Computer Science and Engineering, Korea University, Seoul, 02841 Korea; 10 SAS Institute, Inc, Cary, NC 27513 USA; 11 Department of Computer Science and Engineering, University of Nevada, Reno, 89557 USA; 12 Independent Consultant in Computational Biology, Owkin, Inc., New York, NY 10022 USA; 13 IBM Thomas J. Watson Research Center, Yorktown Heights, New York, 10598 USA; 14 Wellcome Trust Sanger Institute, Hinxton, CB10 1SA UK; 15 Early Clinical Development, IMED Biotech Unit, AstraZeneca, Cambridge, SG8 6EH UK; 16 Oncology, IMED Biotech Unit, AstraZeneca, R&D Boston, Waltham, MA 02451 USA; 17 Department of Genetics and Genomic Sciences, Icahn School of Medicine at Mount Sinai, New York, 10029 USA; 185 Present Address: Institute of Computational Biology, Helmholtz Zentrum München - German Research Center for Environmental Health, Munich, D-85764 Germany; 186 Present Address: Heidelberg University, Faculty of Medicine, Institute for Computational Biomedicine, Bioquant, 69120 Heidelberg, Germany; 18 Department of Electrical and Computer Engineering, Texas A&M University, College Station, 77843 USA; 19 Department of Bioinformatics - BiGCaT NUTRIM Maastricht University, Maastricht, 6229 ER The Netherlands; 20 Division of Molecular Carcinogenesis NKI, Amsterdam, 1066 CX The Netherlands; 21 Faculty of EEMCS, Delft University of Technology, Delft, 2628 CD The Netherlands; 22 Genetics Bioinformatics and Computational Biology Virginia Tech, Blacksburg, VA 24061 USA; 23 Institute for Molecular Medicine Finland FIMM, University of Helsinki, Helsinki, FI-00014 Finland; 24 Bioinformatics Research Center North Carolina State University, Raleigh, NC 27695-7566 USA; 25 The Institute of Cancer Research, London, SW3 6JB UK; 26 Computational Bioscience Research Center (CBRC) KAUST, Thuwal, 23955 Saudi Arabia; 27 King Abdullah University of Science and Technology (KAUST), Thuwal, 23955 Saudi Arabia; 28 Centre for Molecular Informatics, Department of Chemistry, University of Cambridge, Cambridge, CB2 1EW UK; 29 Department of Computer Science Virginia Tech, Blacksburg, VA 24061 USA; 30 Alexandria University, Alexandria, 21500 Egypt; 31 Eshelman School of Pharmacy, University of North Carolina at Chapel Hill, Chapel Hill, 27599 USA; 32 Department of Bioinformatics, Igenomix SL, Valencia, 46980 Spain; 33 Department of Radiology, Stanford University, Drive, Stanford, 94305 USA; 34 Bioinformatics Research Center, Department of Chemistry, Department of Statistics, North Carolina State University, Raleigh, NC 27695 USA; 35 Leloir Institute, Buenos Aires, C1405 BWE Argentina; 36 University of Toronto, Toronto, ON M5S Canada; 37 Unit of Computational Medicine, Department of Medicine, Solna SciLifeLab Center for Molecular Medicine Karolinska Institute, Solna, SE-171 76 Sweden; 38 INSERM U1068, Marseille, 13005 France; 39 Institut Paoli-Calmettes, Marseille, 13009 France; 40 Aix-Marseille University, 13007 Marseille, France; 41 CNRS UMR7258, Marseille, 13273 France; 42 Centre Biological Engineering (CEB), University of Minho, Braga, 4710-057 Portugal; 43 Institut HyperCube, Paris, 92907 France; 44 Computational and Systems Biology, Genome Institute of Singapore, Singapore, 138672 Singapore; 45 Indiana University School of Medicine, Indianapolis, 46202 USA; 46 Center for Integrative Medical Sciences RIKEN Japan, Kobe, Hyogo 650-0047 Japan; 47 Cancer Genomics Netherlands, Utrecht, 3584 CG Netherlands; 48 Computer Laboratory, University of Cambridge, Cambridge, CB3 0FD UK; 49 Constellation Pharmaceuticals, Cambridge, 2142 USA; 50 CIMUS University of Santiago de Compostela, La Coruña, 15001 Spain; 51 Bioinformatics and Computational Biology Unit, Department of Biology, University of Rome Tor Vergata, Rome, 00133 Italy; 52 Department of Molecular and Translational Medicine, University of Brescia, Brescia, 25123 Italy; 53 Department of Medical Epidemiology and Biostatistics, Karolinska Institute, Stockholm, SE-171 76 Sweden; 54 Centre de Regulacio Genomica (CRG), Barcelona Institute for Science and Technology, Barcelona, 08036 Spain; 55 Department of Medicine, University of California, San Diego, La Jolla, CA 92093 USA; 56 Department of Biology, University of Rome Tor Vergata, Rome, 00133 Italy; 57 Department of Computer Engineering, Ege University Turkey, Metro İstasyonu, 35100 Turkey; 58 University of Texas Health Science Center, Houston, 77030 USA; 59 Texas A&M University, Station, College Station, TX 77843 USA; 60 Case Western University, Cleveland, 44106 USA; 61 Princess Margaret Cancer Centre, Toronto, M5G 2C1 Canada; 62 Department of Bio and Brain Engineering, Korea Advanced Institute of Science and Technology (KAIST), Daejeon, 34141 South Korea; 63 Department of Medical Information, Kongju Nationtional University, Gongju, 32588 South Korea; 64 Bio-Synergy Research Center, Daejeon, 02451 Republic of Korea; 65 University of Washington Tacoma, Tacoma, 98402 USA; 66 Departement de Biologie Structurale et Chimie Institut Pasteur Unite de Bioinformatique Structurale CNRS UMR, 3825 Paris, France; 67 Clinical Bioinformatic Area Fundacion Progreso y Salud CDCA Hospital Virgen del Rocio, Sevilla, 41092 Spain; 68 Stanford University, Stanford, 94305 USA; 69 KU Leuven, Leuven, 3000 Belgium; 70 Max Planck Institute for Developmental Biology, Tuebingen, 72076 Germany; 71 Department of Computer Science, Wayne State University Michigan, Detroit, 48202 USA; 72 Department of Obstetrics and Gynecology, Wayne State University Michigan, Detroit, 48202 USA; 73 CERTH-ITI, Thessaloniki, GR 5700 Greece; 74 Institute of Data Science, Maastricht University, Maastricht, 6229 ER Netherlands; 75 Al-Azhar University, Cairo, 11651 Egypt; 76 National Research Centre, Cairo, 12622 Egypt; 77 Nile University, Cairo, 12622 Egypt; 78 Division of Medicinal Chemistry, AIMMS Vrije Universiteit, Amsterdam, 1081 HZ The Netherlands; 79 LaSIGE Faculdade de Ciencias Universidade de Lisboa Portugal, Lisboa, 1749-016 Portugal; 80 University of A Coruna, Coruña, 15001 Spain; 81 Center for Computational Biology and Bioinformatics, University of California, San Diego, La Jolla, CA 92093 USA; 82 Norwegian University of Science and Technology, Trondheim, 7491 Norway; 83 St. Olav’s University Hospital, Trondheim, 7030 Norway; 84 Texas State University, San Marcos Texas, San Marcos, 78666 USA; 85 University of Oxford, Oxford, OX1 2JD UK; 86 Bioinformatics Research Center, Department of Chemistry, North Carolina State University, Raleigh, NC 27695 USA; 87 Oslo Centre for Biostatistics and Epidemiology, University of Oslo and Oslo University Hospital Norway, Oslo, 0317 Norway; 88 Department of Biomedical Sciences, City University of Hong Kong, Hong Kong, 999077 Hong Kong; 89 Yale University, New Haven, 06520 USA; 90 Program for Evolutionary Dynamics, Harvard University, Cambridge, 02138 USA; 91 Institut Curie Inserm U900, Paris, 75248 France; 92 The Systems Biology Institute Tokyo, Tokyo, 141-0022 Japan; 93 Department of Computer Science Aalto University, Espoo, 02150 Finland; 94 Departament d’Enginyeria, Quimica Universitat Rovira i Virgili, Tarragona, 43007 Spain; 95 Sanford Burnham Prebys Medical Discovery Institute, Jolla, 92037 USA; 96 Hospital for Sick Children, Toronto, Ontario, M5G 1X8 Canada; 97 Mucosal and Salivary Biology Division King’s College London Dental Institute, London, SE1 9RT UK; 98 College of Engineering, Koc University, Istanbul, 34450 Turkey; 99 School of Medicine, Koc University, Istanbul, 34450 Turkey; 100 MRC Biostatistics Unit University of Cambridge, Cambridge, CB2 0SR UK; 101 University of Washington, Seattle, WA 98195 USA; 102 ICREA, Barcelona, 08010 Spain; 103 Joint IRB-BSC-CRG Program in Computational Biology Institute for Research in Biomedicine, Barcelona, 08028 Spain; 104 Princess Margaret Cancer Centre, University Health Network, Toronto, M5G 2C1 ON Canada; 105 Ph.D. Program in Computer Science, The Graduate Center, The City University of New York, New York, 10016 USA; 106 Department of Mathematics University of Oslo, Oslo, 0371 Norway; 107 Institute of Machine Learning Johannes Kepler University Linz, Linz, 4040 Austria; 108 University of Wisconsin, Madison, 53706 USA; 109 Department of Tumor Biology Institute for Cancer Research, Oslo University Hospital Norway, Oslo, 0424 Norway; 110 Department of Informatics University of Oslo, Oslo, 0316 Norway; 111 Wellcome Trust Centre for Human Genetics University of Oxford, Oxford, OX3 7BN UK; 112 Bioinformatics and Systems Biology Program, University of California, San Diego, La Jolla, CA 92093 USA; 113 University of Minnesota, Minneapolis, 55455 USA; 114 Department of Computer Science and Statistics, Daegu University, Daegu, 38453 South Korea; 115 Department Bioinformatics, University of Texas Southwestern Medical Center, Dallas, TX 75390 USA; 116 Department of Computer Science, The University of Suwon, Suwon, 16499 Republic of Korea; 117 Department of Biomedical Informatics, Columbia University in the City of New York, New York, 10032 USA; 118 School of Statistics Shandong, University of Finance and Economics, Jinan, 250000 China; 119 Department of Computer Science, Hunter College, The City University of New York, New York, NY, 10065 USA; 120 Department of Biostatistics, School of Public Health Shandong University China, Shandong, 250012 China; 121 Skolkovo Institute of Science and Technology, Moscow, 121205 Russia; 122 A.A.Kharkevich Institute for Information Transmission Problems, Moscow, 127051 Russia; 123 Algorithmic Dynamics Lab Unit of Computational Medicine, Department of Medicine Solna, SciLifeLab Center for Molecular Medicine Karolinska Institute, Solna, SE-171 76 Sweden; 124 Standigm Inc, Seoul, 06250 Korea; 125 Department of Bioengineering, University of Washington, Seattle, 98195 USA; 126 Division of Oncogenomics NKI, Amsterdam, 1066 CX The Netherlands; 127 Department of Genetics, Stanford University, Stanford, 94305 USA; 128 Department of Cancer Biology, Stanford University Stanford, Stanford, 94305 USA; 129 Oslo Centre for Biostatistics and Epidemiology, Department of Biostatistics, University of Oslo, Oslo, 0317 Norway; 130 Helmholtz Zentrum Munchen Institute of Health Economics and Health Care Management, Neuherberg, 85764 Germany; 131 Data Science Radboud University, Nijmegen, 6525 Netherlands; 132 The Ohio State University College of Medicine, Department of Biomedical Informatics, Columbus, 43210 USA; 133 Department of Medicine, University of Cambridge, Cambridge, CB2 0SP UK; 134 Centre for Computational Biology Duke-NUS, Medical School Singapore, Singapore, 169857 Singapore; 135 SingHealth Duke-NUS Institute of Precision Medicine Singapore, Singapore, 169857 Singapore; 136 Carnegie Mellon University, Pittsburgh, 15213 USA; 137 Department of Medical Science, Mathematics Medical Research Institute Tokyo Medical and Dental University, Tokyo, 113-8510 Japan; 138 CREST JST, Tokyo, 102-0076 Japan; 139 Weill Cornell Medicine, New York, NY, 10065 USA; 140 Ghent University, Gent, 9000 Belgium; 141 Bioinformatics Center Institute for Chemical Research Kyoto University, Kyoto, 611-0011 Japan; 142 Helsinki Institute for Information Technology HIIT, Department of Computer Science, Aalto University Finland, Espoo, 02150 Finland; 143 University of Turku, Turku, 20500 Finland; 144 Helsinki University, Helsinki, 00100 Finland; 145 Interuniversity Institute of Bioinformatics in Brussels, Ixelles, 1050 Belgium; 146 Ghent University Bogor Agricultural University, Gent, 9000 Belgium; 147 Institut de Mathematiques de Toulouse, Universite Paul Sabatier, Toulouse, 31400 France; 148 University of Cambridge, Cambridge, CB2 8PQ UK; 149 Texas Tech University, Lubbock, 79409 USA; 150 Structural Bioinformatics Group GRIB IMIM, Department of Experimental and Life Sciences, Universitat Pompeu Fabra Barcelona, Catalonia, 08041 Spain; 151 Machine Learning Group (MLG), Department d’Informatique, Universite libre de Bruxelles (ULB), Brussels, 1050 Belgium; 152 Harvard University, Cambridge, 02138 USA; 153 University of Illinois at Urbana-Champaign Urbana, Champaign, 61820 USA; 154 Microsoft Research Redmond, Redmond, 98052 USA; 155 Cambridge Rindge and Latin High School, Cambridge, 02138 USA; 156 Catalan Institute of Oncology (ICO), Barcelona, 199 08908 Spain; 157 Bellvitge Biomedical Biomedical Research Institute (IDIBELL), Barcelona, 199 08908 Spain; 158 Institut Curie RT2 Lab, Paris, 75248 France; 159 Division of Environmental Health Sciences, School of Public Health, University of California, Berkeley, CA 94720 USA; 160 Division of Molecular Pathology NKI, Amsterdam, 1066 CX The Netherlands; 161 Kyungpook National University, Daegu, 41566 South Korea; 162 FOM University of Applied Sciences, Essen, 45127 Germany; 163 Institute for Integrated and Intelligent Systems Griffith University, Mount Gravatt, QLD 4111 Australia; 164 University of the South Pacific Fiji, Suva, Fiji; 165 Department of Electrical Electronic and Computer Engineering, Gifu University, Gifu, 501-1193 Japan; 166 Center for Statistics, Hasselt University Belgium, Hasselt, 3590 Belgium; 167 Faculty of Medicine, University of British Columbia, Kelowna, V1Y 1T3 Canada; 168 Center for Quantitative Genetics and Genomics, Department of Biology and Genetics, Aarhus University, Aarhus, 8000 Denmark; 169 Technische Universitat Munchen, München, 80333 Germany; 170 Department of Computer Science, National University of Singapore, Singapore, 117417 Singapore; 171 Research Unit on Biomedical Informatics, University Pompeu Fabra UPF, Barcelona, 08041 Spain; 172 Biological and Environmental Science and Engineering, Division KAUST, Thuwal, 23955 Saudi Arabia; 173 Cancer Hospital of Chinese Academy of Sciences, Hefei, 230031 China; 174 Drug Discovery and Safety Leiden, Academic Centre for Drug Research, Leiden University, Einsteinweg 55, Leiden, 2333 CC The Netherlands; 175 Department of Neurosurgery, Cancer Center, Amsterdam, 1012 WX The Netherlands; 176 Department of Computer Science, Hunter College and The Graduate Center, The City University of New York, New York, NY 10016 USA; 177 Hematology Research Unit Helsinki, Department of Clinical Chemistry and Hematology, University of Helsinki and Helsinki University Hospital Comprehensive Cancer Center, Helsinki, 00029 Finland; 178 Institute of Cancer and Genomic Sciences, University of Birmingham, Birmingham, B15 2SY UK; 179 University of California, San Diego, SD, 92093 USA; 180 National Center for Advancing Translational Sciences National Institutes of Health, Rockville, MD 20892 USA; 181 Universidad Politecnica de Madrid, Madrid, 28040 Spain; 182 Oxford Immune Algorithmics Ltd, Oxford, RG1 3EU UK; 183 Indiana University - Purdue University, Indianapolis, 47907 USA; 184 Department of Electronic Engineering, City University of Hong Kong, Hong Kong, 999077 Hong Kong

**Keywords:** Machine learning, Statistical methods, High-throughput screening, Systems biology, Cancer

## Abstract

The effectiveness of most cancer targeted therapies is short-lived. Tumors often develop resistance that might be overcome with drug combinations. However, the number of possible combinations is vast, necessitating data-driven approaches to find optimal patient-specific treatments. Here we report AstraZeneca’s large drug combination dataset, consisting of 11,576 experiments from 910 combinations across 85 molecularly characterized cancer cell lines, and results of a DREAM Challenge to evaluate computational strategies for predicting synergistic drug pairs and biomarkers. 160 teams participated to provide a comprehensive methodological development and benchmarking. Winning methods incorporate prior knowledge of drug-target interactions. Synergy is predicted with an accuracy matching biological replicates for >60% of combinations. However, 20% of drug combinations are poorly predicted by all methods. Genomic rationale for synergy predictions are identified, including ADAM17 inhibitor antagonism when combined with PIK3CB/D inhibition contrasting to synergy when combined with other PI3K-pathway inhibitors in PIK3CA mutant cells.

## Abstract (web summary)

Resistance to first line treatment is a major hurdle in cancer treatment, that can be overcome with drug combinations. Here, the authors provide a large drug combination screen across cancer cell lines to benchmark crowdsourced methods and to computationally predict drug synergies.

## Introduction

Personalized treatment matching targeted drugs to a tumor’s genetics has resulted in remarkable responses. Unfortunately, most patients’ tumors develop resistance leading to disease relapse. There are multiple mechanisms that may lead to drug resistance^1^ that include genetic and non-genetic heterogeneity inherent in advanced cancers, coupled with complex feedback and regulatory mechanisms, and dynamic interactions between tumor cells and their microenvironment. Any single therapy may be limited in its effectiveness, but drug combinations are hypothesized to potentially overcome drug resistance and lead to more durable responses in patients. The molecular makeup of cancer cells and the mechanisms driving resistance will influence the optimal combination of mechanisms to target^1–3^.

High-throughput preclinical approaches are crucial to determine and evaluate effective combination strategies. While empirical experiments are important for observing potential synergistic properties across drug pairs, the possible number of combinations grows exponentially with the number of drugs under consideration. This is further complicated by the influence of disease and cellular contexts, rendering it impractical to cover all possibilities with undirected experimental screens^4^. Computational approaches for predicting drug synergy are critical to guide experimental approaches for discovery of rational combination therapy^5^.

A number of approaches have been developed to model drug combination synergy using chemical, biological, and molecular data from cancer cell lines^6,7^ but with limited translatability to the clinic. A key bottleneck in the development of such models has been a lack of public data of sufficient size and variety to train computational approaches^4,8^, particularly considering the diversity of biological mechanisms that may influence drug response. A further limit to the translatability of many computational approaches is their reliance on data features that may not be present during the course of patient care, such as on-treatment tumor molecular profiles.

To accelerate the understanding of drug combination synergy, Dialog for Reverse Engineering Assessments and Methods (DREAM) Challenges partnered with AstraZeneca and the Sanger Institute to launch the AstraZeneca-Sanger Drug Combination Prediction DREAM Challenge. DREAM Challenges (dreamchallenges.org[www.dreamchallenges.org]) are collaborative competitions that pose important biomedical questions to the scientific community, and evaluate participants’ predictions in a statistically rigorous and unbiased way, emphasizing model reproducibility, and methodological transparency^9^.

This Challenge was designed to explore fundamental traits that underlie effective combination treatments and synergistic drug behavior. Specifically, it was structured to address the following translational questions using data available prior to drug treatment (mirroring a clinically relevant scenario to direct therapeutic choice): [i] how to predict whether a known (previously tested) drug combination will be effective for a specific patient; [ii] how to predict which new (untested) drug combinations are likely to yield synergistic behaviors in a patient population; and [iii] how to identify novel biomarkers that may help reveal underlying mechanisms related to drug synergy.

We shared with the scientific community 11,576 experimentally tested drug combinations on 85 cancer cell lines. Molecular data was provided for the untreated (baseline) cell lines, alongside chemical information for the respective drugs. Participants used the described data to train and test models, and were encouraged to extend computational techniques to leverage a priori knowledge of cellular signaling networks.

In this manuscript, we report on the results of this Challenge where we have identified novel and performant methods using a rigorous evaluation framework on previously unpublished data. We describe the details of these approaches, as well as general trends arising from the meta-analysis of all submissions. The full dataset, along with methods and scoring functions, are freely provided to the research community, and available to benchmark future algorithms in the field. Finally, we describe putative mechanistic models derived from the observed predictive features underlying synergistic responses, particularly between receptor tyrosine kinase and PI3K/AKT pathway inhibitors.

## Results

### A large high-throughput drug combination screen

**Fig. 1:**
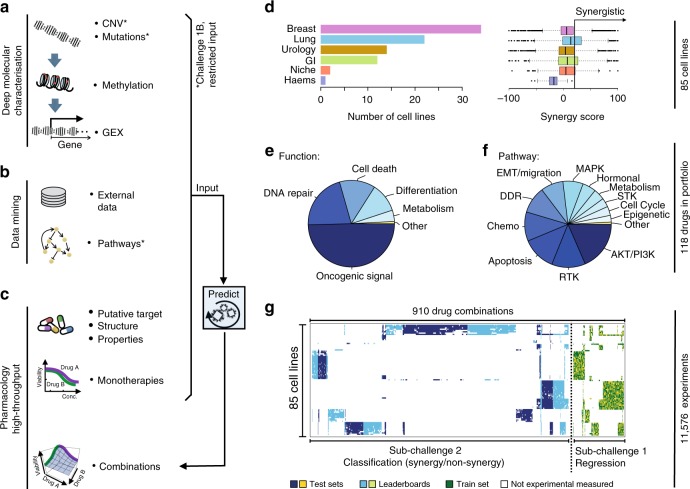
Drug combinations and cell lines profiled. **a** Molecular characterization of the cell lines included genetics, epigenetics, and transcriptomics. **b** Participants were encouraged to mine external data and pathway resources. **c** Participants were provided the putative targets for all and chemical structures for ~$${\raise0.5ex\hbox{$\scriptstyle 1$}\kern-0.1em/\kern-0.15em \lower0.25ex\hbox{$\scriptstyle 3$}}$$ of drugs (with this manuscript structures are now provided for all drugs). **d** The cell line panel contained 85 cell lines from six different cancer types, combination synergy scores capped within a range of −100 to 100. **e** The drug portfolio comprised approximately half oncogenic signaling targeting agents, and half cytotoxic drugs of which 14 were untargeted chemotherapies. **f** Drugs split by the putative targeted pathway. **g** Sparse data was split into training set, leaderboard, and independent test set for SC1 and SC2

We collated a combinatorial drug sensitivity screen comprising 11,576 experiments each measured in a 6-by-6 dose matrix (Methods) across 85 cancer cell lines. A synergy and an antagonism distribution (Loewe reference model^10,11^) were calculated and summarized via a single score for each experimental matrix (Methods; Supplementary Fig. 1 and Supplementary Data 1). The resulting dataset included highly reproducible cell viability response measurements and synergy scores for 910 pairwise combinations of 118 drugs (Supplementary Fig. 2 and Methods), plus information on the drugs including putative drug targets and their chemical properties. We also integrated deep molecular characterization of these same cell lines, including somatic mutations, copy-number alterations, DNA methylation, and gene expression profiles (Fig. 1a–c) measured before drug treatment^12^.

The 85 cell lines were predominantly derived from tumors of the breast (*N* = 34), lung (*N* = 22), bladder (*N* = 14), and the gastrointestinal tract (*N* = 12) (Fig. 1d). Drug synergy score distributions varied across disease types (Fig. 1d); in particular lung cell lines had over twofold higher mean synergy than breast cell lines (*t*-test *P* = 7*e*-27). Of the 118 drugs tested, 59 were targeted therapies against components of oncogenic signaling pathways (Methods), 15 of which target receptor tyrosine kinases (RTKs), 22 target PI3K/AKT signaling, and 9 target MAPK signaling (Fig. 1e, f). Across the pairwise drug combination experiments, 88% (*N* = 797) of the unique pairs had drug targets within the same signaling pathway and demonstrated markedly overall higher synergy scores (average of 17.3 vs. 7.3, *t*-test *P* = 2*e*–18) than the remaining 12% (*N* = 113) whose drug targets were defined to be in distinct pathways. As part of the Challenge design, we ensured that drug targeted pathways and cancer types were proportionally distributed across sub-challenges and training/test datasets (Fig. 1g). Sparsity in the cell line drug combination data matrix (Fig. 1g) resulted as several drug combinations were selectively profiled in clinically relevant cancer cell lines, e.g., ESR1 inhibitors were predominantly combined with other drugs in estrogen receptor-positive breast cancer cell lines since these agents are standard of care within this cancer subtype.

### Comparison of AZ-DREAM to published combination screens

**Fig. 2:**
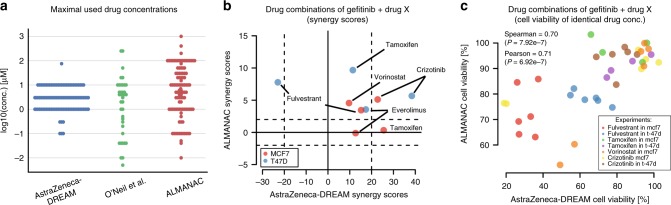
Reproducibility of independent in vitro drug combination datasets. **a** Comparison of the maximum drug concentration used in the AZ-DREAM, O’Neil et al.^4^ and ALMANAC datasets. **b** Synergy scores of identical drug combinations in AZ-DREAM and ALMANAC. Horizontal and vertical striped lines mark the threshold for synergistic and antagonistic, where positive and negative scores confirm synergy and antagonism, respectively. **c** Shows cell viability for each of the 38 overlapping experimental titration points that are identical in AZ-DREAM and ALMANAC across the nine drug combination experiments, significance calculated with test for association (Supplementary Fig. 3)

We compared the AstraZeneca-DREAM (AZ-DREAM) challenge dataset with the independently published in vitro combination screening studies by O’Neil et al.^4^ and ALMANAC^13^. All three studies used different experimental designs and protocols (Fig. 2a and Supplementary Table 1), with AZ-DREAM exploring screens with a 5-by-5 concentration titration format, O’Neill et al.^4^ a 4-by-4 format, and ALMANAC a 3-by-3 format. The overlap is limited in cell lines, targets and drugs explored: 10 cells and 7 targets, 4 drugs and 0 combination-cell pairs shared between AZ-DREAM and O’Neil et al.^4^; and 15 cells, 28 targets, 19 drugs, and 10 combination-cell pairs shared between AZ-DREAM and ALMANAC. AZ-DREAM provides greater coverage of targeted agents and greater numbers of cell lines per tumor type (Supplementary Table 1).

For the AZ-DREAM and ALMANAC comparison, only nine experiments passing quality control (Supplementary Methods) had the same combination tested in the same cell line, comprising gefitinib combined with either tamoxifen, fulvestrant, vorinostat, crizotinib, or everolimus, and tested in two breast cancer cell lines (MCF7 or T47D). Encouragingly, all but one experiment were concordantly identified synergistic with the same effect signs seen in AZ-DREAM and ALMANAC (Fig. 2b). Within the nine overlapping experiments, 38 titrations were identically used by both ALMANAC and AZ-DREAM (Supplementary Fig. 3 and Supplementary Fig. 4a). For these 38 titrations we observed a correlation >0.7 (Fig. 2c and *P* < 10^−7^, test for association) between cell viability measures from AZ-DREAM and ALMANAC, maintained for individual combinations and cell lines where the number of data points was sufficient (Supplementary Fig. 4).

While there were ten cell lines and four drugs found in both AZ-DREAM and O’Neil et al.^4^, no combination-cell experiment was shared between those two screens. O’Neill et al.^4^ data displayed lower dispersion of synergy scores (Supplementary Fig. 2c, d) and fewer instances of extreme synergy scores. The technical reproducibility within these screens, however, was comparable, with a similar correlation observed between replicated experiments (Spearman = 0.63 for O'Neil et al.,^4^ Spearman = 0.56 for AZ-DREAM).

We derived a RECIST-like response measure from the AZ-DREAM in vitro data (see Methods) to enable comparison to in vivo response metrics for the eight overlapping combinations in patient-derived tumor xenograft (PDX) models published by Gao et al.^8^ (Supplementary Data 1 and Supplementary Fig. 5a). We compared the % PDX models with synergy in Gao et al.^4,8^ to the % cell lines with synergy in AZ-DREAM. A concordant trend was observed (Pearson *r* = 0.34, *P* = 0.42; Methods; Supplementary Fig. 5b), although too few combinations were shared between the datasets to conclude with statistical confidence.

### Benchmarking of methods reveal high prediction accuracy

The Challenge was divided into two primary sub-challenges. In sub-challenge 1 (SC1) participants were asked to predict continuous synergy scores for drug combinations for which training data on those same combinations were available. In sub-challenge 2 (SC2), participants were asked to predict binary synergy status on drug combinations for which no training data was provided, thereby requiring participants to infer synergy using transferable data/knowledge patterns identified from previously seen independent drug pairs. SC1 was further subdivided into two parts: SC1A allowed the use of all available data for model prediction, while SC1B limited data use to just mutation and copy-number variation (mimicking current clinical assay feasibility).

A total of 969 participants of diverse geography and expertize registered for the Challenge (Supplementary Fig. 6a, b). One-hundred sixty teams submitted across any portion of the Challenge and 78 teams submitted for final assessment. Specifically, SC1A received final submissions from 76 teams, 62 for SC1B and 39 for SC2.

**Fig. 3:**
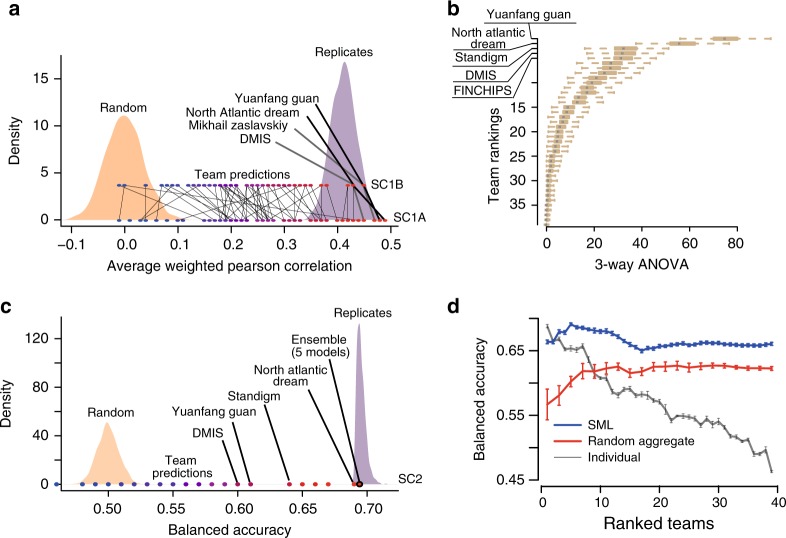
Performance of teams in the AstraZeneca-DREAM challenge. **a** Participant performance in SC1A and SC1B— the distribution of performance of random predictions was used to estimate a lower limit, and the distribution of synergy correlations between biological replicates were used to estimate the upper limit. **b** Participant performance ranked in SC2 based on the primary metric, 3-way ANOVA. Distribution of bootstrap prediction performances for each team are shown by each boxplot with the dot showing their actual performance. **c** Participant performance plotted with upper and lower limits for SC2 based on the tie-break metric. **d** Ensemble models compared to the performance of individual models ranked from best to poorest performing in SC2. SML is an ensemble of the best performing models based on estimation of their BAC. Random Aggregation is an ensemble combining a random combination of models. Standard error of mean represented by error bars are estimated from ten random splits of the data

As scoring metric we used the average weighted Pearson correlation between the continuous endpoints of predicted and known synergy values for SC1, and both the *–*log_10_(*p*) from a 3-way analysis of variance (ANOVA) and balanced accuracy (BAC) for SC2 where predictions were binary (Methods). Across all teams, mean performance scores were *r* = 0.24 ± 0.01 and *r* = 0.23 ± 0.01 (weighted Pearson correlation ± standard error) for SC1A and SC1B, respectively, and –log10(*p*) = 12.6 (3-way ANOVA) for SC2. Despite omitting several input data types, teams performed only slightly worse for SC1B, Δprimary metric = 0.01 (*t*-test *P* = 0.90), compared to SC1A (Fig. 3a and Supplementary Fig. 6c, d). While teams employed many different methodological approaches to modeling drug synergy—including regression, decision trees, random forests, Gaussian processes, SVM, neural networks, text mining, mechanistic network-based, and others (Supplementary Fig. 7a)—algorithm class showed little relationship to performance (Supplementary Fig. 7b). The top winning team in all three sub-challenge was *Yuanfang Guan* (Y Guan) with primary metrics of 0.48, 0.45, and 74.89 in SC1A, SC1B, and SC2, respectively. Based on the primary metric in SC2, Y Guan performed considerably better (Methods; >5 Bayes Factor, based on bootstrapped metrics’ comparisons) than other teams (Fig. 3b). All performance statistics and team rankings are available at the Challenge website (synapse.org/DrugCombinationChallenge[https://www.synapse.org/#!Synapse:syn4231880/wiki/235649]).

To benchmark the performance of teams in the final rounds of SC1A/B and SC2, we established lower and upper bounds of performance. We defined the lower bound as the null model, i.e., random permutation of the synergy data across each cell line (see ‘Code availability’’ section). We would not expect algorithms to predict better than the reproducibility observed between replicate experiments. We therefore identified cases for which replicate measurements (same drug/combination tested independently in the same cell line) were available and assessed the primary metric achieved when using one measure to predict the other. We set this metric value as our upper-bound. We observed that 83%, 85%, and 94% of submitted models performed better than random (Methods; 5% false discovery rate, FDR) for SC1A, SC1B, and SC2, respectively. Team performances varied widely, but remarkably the top 15 models (20%) submitted to SC1A all reached a performance level comparable to experimental replicates (primary metric = 0.43; Fig. 3a), as did the top 13 models (21%) in SC1B. Proportionally fewer teams performed at the level of replicate experiments in SC2 based on the BAC, with North Atlantic Dream (NAD) achieving the best performance (BAC = 0.688; Fig. 3c).

Given the less robust performance of SC2, we assessed whether an ensemble method—based on an aggregation of all submitted models—could yield a better overall model, a phenomenon called “wisdom of the crowd”^9,14^. By applying a Spectral Meta-Learner (SML)^15^ as our ensembl approach, we achieved a modest improvement in performance (BAC = 0.693) over the best performing individual team (BAC = 0.688), as well as an ensemble of randomly selected models (BAC = 0.63, (Fig. 3d).

### Leveraging biological relationships improves predictions

A common strategy among top-performing teams (DMIS, NAD, and Y Guan) was to filter molecular features, leaving only those related to known cancer drivers for subsequent modeling (Supplementary Methods). These teams also consolidated pharmacological and/or functional pathway information associated with the molecular drug target, enabling one drug’s model to learn from data generated for another drug with the same target (Y Guan^16^ and NAD^16–18^).

**Fig. 4:**
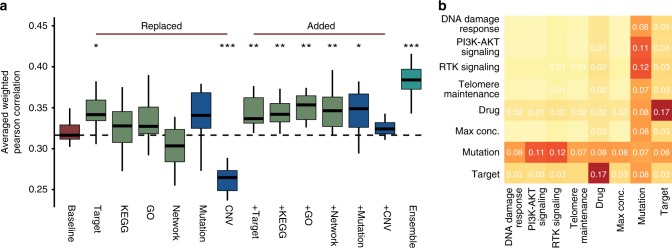
Feature impact. Drug target annotation is key in top-performing algorithms, as is the meta information about variants including their functional impact and tumor driver gene status. **a** Cross validation-based distributions of NAD primary metric of SC1B when replacing or adding drug/cell line label with respective features. NAD baseline model (red) used cell line labels and drug labels only as feature inputs. In the other models different drug specific (drug targets, drug target KEGG pathway memberships, drug target-associated Gene Ontology terms or direct interactions between drug targets in a signaling network) or cell line specific (mutations or CNVs of selected, cancer related genes) features (green and blue, respectively) were added either in place of or in addition to the baseline model features. Ensemble model (cyan) is the averaged prediction of the different models. Single asterisks refers to *t*-test *P* < 0.05, double asterisks for *P* < 0.01, and triple asterisks for *P* < 0.001 compared to baseline model. **b** Heatmap of decrease in performance (average weighted Pearson correlation) of SC1B for DMIS support vector regression method when a particular feature type is removed (diagonal) or two feature types are removed at once (off diagonal)

We took two approaches to analyze each feature type’s importance, particularly whether incorporating molecular features and chemical/biological knowledge can increase prediction accuracy. In the first approach, we generated a baseline model (following Team NAD’s method) utilizing only cell line and drug labels as input features as per SC1B. We then assessed changes in the primary metric after the substitution or addition of feature types (Fig. 4a and Supplementary Methods). In the second approach, we started with the DMIS model obtained from SC1B, and then iteratively removed single feature types and pairs of features to assess changes in prediction accuracy (Fig. 4b and Supplementary Methods).

Surprisingly, high primary metrics were observed for the NAD baseline model where the only input features were drug and cell line label (Fig. 4a, 0.32). Drug target was the only feature to improve performance of the NAD baseline model when swapped with drug label (Fig. 4a, *t*-test *P* = 0.012). Furthermore, removing both drug label and target resulted in the highest performance drop for the DMIS model (Fig. 4b, −0.17). This result highlights the importance of the global cell-line state in predicting drug synergy, and how drug target information shared across drugs can facilitate transfer learning across separate models. Mutational and copy-number variation (CNV) data can similarly offer a barcode of cell identity to encode cell line label. However, where mutation data improved performance when replacing cell line labels, replacement with CNV decreased performance significantly (Fig. 4a, *t*-test *P* = 8.8e-6). Importantly, in all cases additional feature data increased performance when added to the NAD baseline model (Fig. 4a, *t*-test *P* = 0.009, 0.009, 0.002, 0.008, 0.021 adding drug target, KEGG pathway, Gene Ontology, signaling network, and mutation features, respectively). Ensemble of different feature sets improved prediction most when collectively increasing coverage of biological (pathway) complexity (Fig. 4a, *t*-test *P* = 1.2e-6).

### Inspecting consistently poorly predicted drug combinations

**Fig. 5:**
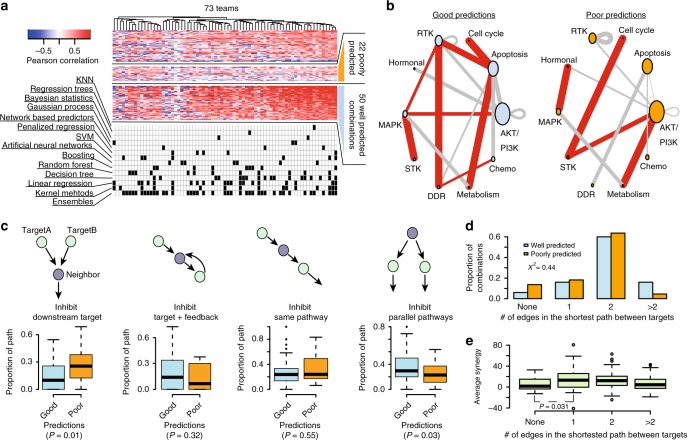
Features of poorly and well predicted combinations. **a** Heatmap of Pearson correlation between observed and predicted synergy scores for 118 combinations across 73 teams participating in SC1A/B. Algorithms used by each team is marked in the matrix below. **b** Combinations of pathways targeted. Size of node is proportional to number of drugs targeting a specific pathway in the entire dataset and width of edges is proportional to the number of drug combinations within the poorly or well predicted combination subset. Red edges highlight target pathway combinations unique to either the well predicted or poorly predicted combinations. **c** Types of interactions between the nearest neighboring gene and the two drug targets of poorly and well predicted combinations. Boxplots show the difference in the proportion of interactions of each type for poorly and well predicted combinations (*t*-test). **d** Proportion of poorly and well predicted combinations for different network distances (minimum number of interactions in the OmniPath shortest path) between the two targets of a drug combination, difference significance estimated with chi-squared test. **e** Difference in average synergy for combinations categorized by the network distance between targets are tested with *t*-test

While a global performance metric applied to all cell-lines and drug combinations provides a broad assessment of model prediction accuracy, we hypothesized that some models may be optimized for certain subclasses of combinations and/or tumor types. We assessed the Pearson correlation between predicted and observed synergy scores for each combination in SC1A/B, and clustered teams by correlation of performance across combinations. Of the 118 combinations that had observed synergy scores >20 in more than one cell line, we identified 22 combinations predicted poorly by all participants (Fig. 5a, Methods), and over 50 combinations predicted well across all teams.

Combinations tested across a higher diversity of tumor types tended to show lower overall performance (Supplementary Fig. 8a; *t*-test *P* = 0.04), indicating that a pan-cancer prediction presents a more difficult prediction task. Combinations tested across more tumor types were also tested across greater numbers of experiments (Supplementary Fig. 8b; Spearman = 0.56, *P* = 2.3e–15), but no significant difference was observed between performance and number of experiments within a cancer type specific setting (Supplementary Fig. 8c, d). On average the quality assessment scores were significantly better (*t*-test *P* = 0.018) for the pharmacology experiments in the training set of well predicted compared to poorly predicted combinations (Supplementary Fig. 8e). Comparable trends were seen between the quality of synergy and predictive performance in the training and test sets (Supplementary Fig. 8f; *r* = 0.52 vs. 0.43). The distribution of synergy scores were similar between poorly and well predicted combinations (Supplementary Fig. 8g) as were the proportion of synergistic cases (37% for poorly predicted vs. 39% for well predicted).

Well predicted cases were enriched for combinations inhibiting both the PI3K/AKT and MAPK pathways (Fig. 5b, average Pearson *r* = 0.37 vs. 0.25; *t*-test *P* = 0.008), or apoptosis pathway combined with either metabolism, cell cycle, or receptor tyrosine kinase pathways. The drugs targeting these pathways were prevalent in our dataset, but these specific combinations of those drugs were not (Supplementary Fig. 9a). Assessment of the interactions between drug targets and neighboring proteins from *OmniPath*, a comprehensive compendium of literature-based pathway resources^19^, revealed no differences in the somatic alteration frequency for targets or their first neighbors between the poorly and well predicted combinations (Supplementary Fig. 9b, c). We did observe a significant enrichment of well predicted combinations where both drugs’ respective targets were downstream of a common neighboring protein (Fig. 5c, *t*-test *P* = 0.01), and conversely, we observed an enrichment of poorly predicted combinations where targets were both up-stream (Fig. 5c, *t*-test *P* = 0.03). There was no significant difference (Chi-sqr *P* = 0.44) in OmniPath protein network distance between drug targets for well and poorly predicted combinations (Fig. 5d), nor any correlation between either network distance and average/median synergy scores (Fig. 5e) or the number of cases with synergy >20. Combinations where targets were found to not be connected in a protein network had significantly lower average synergy (*t*-test *P* = 0.031) and lower max synergy (*t*-test *P* = 0.0021).

### Biomarkers of drug combination synergies

A limitation of many machine learning algorithms is the lack of feature interpretability and experimentally testable logic-based rules. We took two approaches to identify biomarkers that may be predictive of drug synergies: a direct survey of participants through which predictive features were nominated for each drug pair (Supplementary Data 2); and retrospective work focusing on results from two of the best performing teams, NAD and DMIS, to deconvolute features most impactful to model predictions (Supplementary Fig. 10 and Supplementary Data 3).

**Fig. 6:**
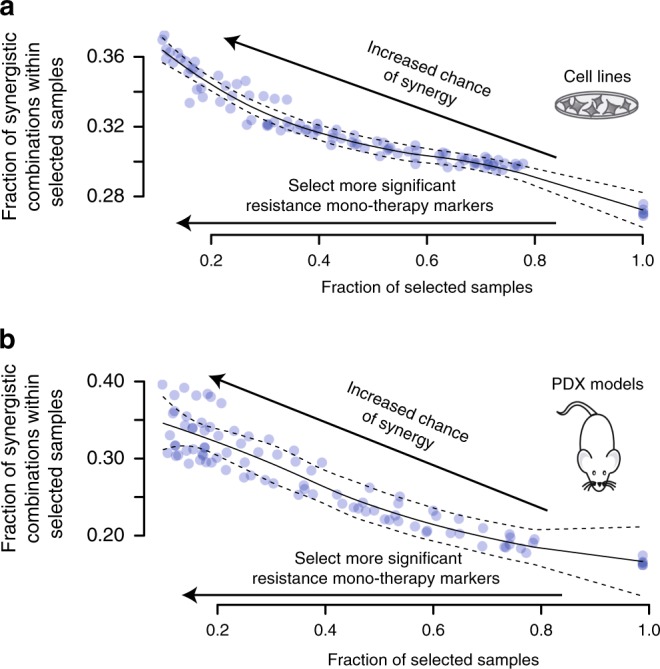
Drug synergy and monotherapy resistance biomarker relationship. **a** Cell lines and **b** PDX models show increased frequency of synergistic drug combinations if they contain biomarkers with stronger association to monotherapy resistance (Methods; Supplementary Methods). The fraction of combination experiments is selected by order of most significant linked monotherapy resistance biomarker associations. The solid and dashed line are the LOcal regrESSion (LOESS) curve fit and its standard error, respectively

The survey-submitted biomarker results varied in detail and depth (Supplementary Data 2), but common genetic markers were apparent across good predictions in SC1B, including *EGFR*, *ERBB2*, *PIK3CA, PTEN*, *TP53*, or *RB1*. In the survey, synergy was commonly assigned to drug pairs targeting directly down- or up-stream of a mutated, amplified, overexpressed or deleted cancer gene. We hypothesized that drug synergy may result when one drug overcomes a resistance mechanism for the other. Focusing on mutations in cancer genes (as defined by Iorio et al.^12^) we identified all mutations associated with resistance to monotherapy in our data (Supplementary Methods and Methods) selected at increasingly stringent *P*-value (wilcoxon rank sum test) thresholds (Supplementary Methods and Methods). For each threshold, we then assessed the likelihood of synergy seen from combinations paired to these monotherapies in the presence vs. absence of the respective mutation. We observed an increase in the proportion of synergistic drug combinations with each increase in threshold stringency (Fig. 6a, Pearson *r* = −0.90, *P* = 4.09e-38). We observed the same trend in patient-derived xenograft (PDX) models (Fig. 6b, Pearson *r* = −0.95, *P* = 2.2e-49). This observation supports the notion that drug sensitivity may be restored with drug combinations targeting a resistance driver.

**Fig. 7:**
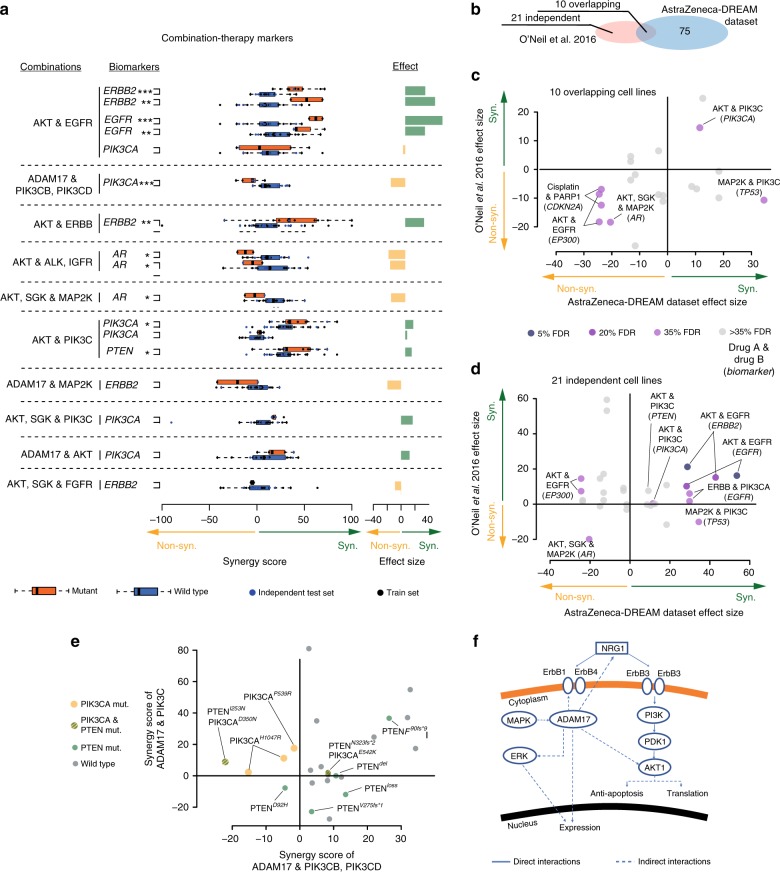
Post-hoc analysis of putative synergy biomarkers. **a** Synergy markers suggested by DMIS and NAD, when focusing on top weighted features from predictive models filtered for biological relatedness to drug targets, ANOVA with FDR of triple asterisks refers to 5%, double asterisks refers to 20% and single asterisks to 35%. **b** Venn-diagram showing independent and overlapping cell lines between AZ-DREAM and O’Neil et al.^4^. Independent dataset reproducibility of biomarker predictions in **c** the overlapping and **d** O’Neil et al.^4^ exclusive cell lines. The effect size is the mean difference in synergy scores of mutant vs. wild-type cell lines. **e** Comparison of ADAM17 combined with PIK3CB/D against ADAM17 in combination with pan-PIK3C inhibitor. **f** Network cartoon of PI3K signaling and role of ADAM17

We also explored models of best performing teams and their chosen features, focusing on biomarker associations aligned to combinations for which the respective team had achieved a robust prediction accuracy (Pearson *r* > 0.5), with particular interest in the genetic biomarkers revealed through SC1B. Multiple criteria for quality, independence and reproducibility (Methods)^4,8^ were applied yielding 13 feature-to-combination associations (Fig. 7a and Supplementary Data 3), seven associated with synergy and six with non-synergy. To assess whether these associations could be independently validated as synergistic biomarkers, we explored nine overlapping and 21 non-overlapping (independent) cell lines in O’Neil et al.^4^ (Fig. 7b) that were treated with similar drug combinations, i.e., same putative drug targets. Concordance of association was observed in the nine overlapping cell lines (Fig. 7c; six out of seven associations, ~86%) and in the 21 independent cell lines (Fig. 7d; eight out of eleven associations, ~72%).

Among the prioritized feature-to-combination associations were several genetic variants associated with synergistic responses to the combination of receptor tyrosine kinase (RTK) inhibitors with inhibitors of the downstream PI3K/AKT pathway. Amplifications or activating mutations in *EGFR* or *ERBB2* consistently predicted synergy from inhibition of the RTK + PI3K/AKT pathways across multiple independent drugs and datasets (Fig. 7a). Less direct relationships were also observed, including combined AKT inhibition with either IGFR inhibition in the *ERBB2* mutant setting or FGFR inhibition in the *EGFR* mutant setting (Supplementary Data 3). Despite kinase domain homology it is unlikely these observations are explained by off-target effects since *EGFR*, *ERBB2*, and *FGFR* mutations were only predictive of respective monotherapy responses (Supplementary Fig. 11). Combinations inhibiting multiple points within the PI3K/AKT pathway also showed synergy in the presence of up-stream activation from mutations in *PIK3CA* or deleterious events in *PTEN* (Fig. 7a, e). Inhibition of the metalloproteinase ADAM17, known to influence RTK activity^20^, also showed synergistic responses in a common subset of cell lines when combined with inhibitors of PIK3C-pan or AKT1/2 (Fig. 7a and Supplementary Data 3), with a notable exception of PIK3CB/D selective inhibitors, which show antagonism unique to *PIK3CA* mutant cell lines (Fig. 7e, f). Amplification and activating mutations in Androgen Receptor (*AR*) were also found to be associated with antagonistic effects when targeting AKT in combination with MAP2K or IGFR inhibitors (Fig. 7a).

### Translatability of synergy and biomarker predictions

**Fig. 8:**
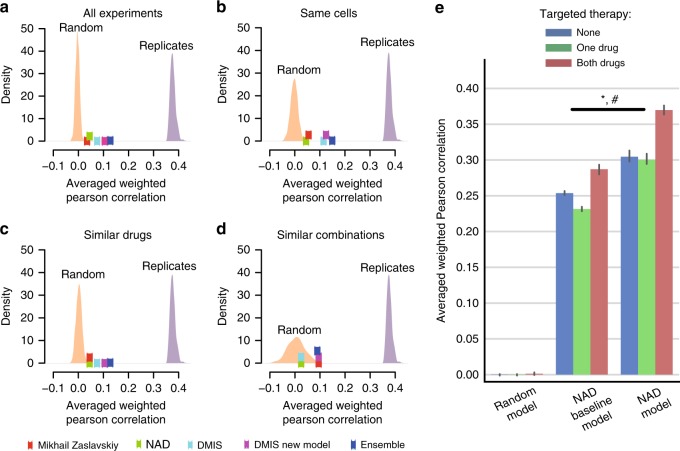
Translatability of AstraZeneca-DREAM models to the independent screens. Performance of SC1A models for predicting synergy scores in the O'Neil et al.^4^ dataset by the best performing teams are plotted along with distributions of predictions from the random model and replicate experiments. Performance of predictions are shown for **a** all experiments in the O’Neil et al.^4^ dataset, and three subsets of the dataset; **b** experiments that tested same cell lines as AZ-DREAM, **c** tested similar drugs as in AZ-DREAM (one drug in the combination with the same target), and **d** tested similar combinations as in AZ-DREAM (same targets for both drugs in the combination). **e** Prediction performance on the ALMANAC^13^ dataset is shown for a random, NAD baseline (using only cell line and drug identities as features) and full NAD model for no targeted agents (None, i.e., two chemotherapeutic drugs), one targeted agent plus chemo (One drug), and combinations of two targeted agents (Both drugs)(mean +/− 95% CI). ANOVA *P*-values: *P = 1.322e-31 for different performance between baseline and full model; #: *P* = 5.584e-5 for association between model performance and number of targeted drugs in the combinations

We assessed the performance of top-performing AstraZeneca-DREAM models on the independent screening datasets by O’Neil et al.^4^ and ALMANAC^13^. Since no combination-cell experiments directly overlapped AZ-DREAM and O’Neil et al.^4^, we collapsed drugs by shared targets to expand the overlap. We observed that SC1A models from NAD and DMIS outperformed random models (Fig. 8a, mean primary metric = 0.07, top 1% of random models) for cell lines and drug target combinations non-overlapping between O’Neill et al. and AZ-DREAM data (Supplementary Data 1). Interestingly, no substantial performance increase was observed when independent model predictions were made for the ten cell lines in common between the two datasets, nor the 30 combinations with similar chemical/target properties (Supplementary Table 2 and Fig. 8b–d). As in the main Challenge, combining these two or more models in an ensemble led to an improved prediction performance (Fig. 8a–d).

Considering the limited overlap and correlation of synergies between AZ-DREAM and ALMANAC datasets, high performance was not expected when predicting ALMANAC synergy scores with models trained on AZ-DREAM data points alone, as was observed for the NAD model. We trained two NAD models on 50% of the ALMANAC data: first a baseline model to show maximum performance achievable when using only cell and drug label features as input; and a second allowing use of the full feature set as input. The full model consistently predicted significantly better than baseline in the remaining 50% over ten randomized iterations, giving confidence in the transferability of the method designed for AZ-DREAM. Best performance was observed for targeted combinations (*r* = 0.369 vs. 0.287 for full NAD and baseline NAD model, ANOVA *P* = 1.322e-31 for model type and *P* = 5.5*e*-05 for model type and targeted therapy association, Fig. 8e).

Exploring AZ-DREAM biomarker associations prioritized as described in earlier sections, we assessed statistical association for drug combinations with consistent targets in the O’Neil et al.^4^ dataset. In the ten cell lines overlapping the AZ-DREAM and O’Neil et al.^4^ datasets, seven of the prioritized biomarker-drug combinations were present, of which six (86%) showed reproducible directionality (FDR < 35%, Fig. 7c). In the 21 O’Neil et al.^4^ cell lines not used within AZ-DREAM training, 11 of the prioritized biomarker-drug combinations were present, of which 8 (72%) showed reproducible directionality (Fig. 7d).

## Discussion

The objective of this AstraZeneca-DREAM Challenge was to drive the development of innovative computational approaches to predict novel drug combinations and to comprehensively benchmark these approaches. To enable this we made one of the largest in vitro drug combinatorial screens to date available to the scientific community. We cover largely non-overlapping experiments to existing datasets and, in particular, offer extensive data for targeted therapies complimenting the non-targeted chemotherapeutic agents covered in the NCI-ALMANAC. Despite little overlap we demonstrated an encouraging reproducibility of data and predictions between screens, particularly when considering the confounding differences in experimental designs and assay formats^21^. Furthermore, we showed that some trends represented in these data can be reproduced in vivo, and that clinically efficacious combination pairs can be identified. Our double-blinded benchmark of 78 methods provides an unbiased comprehensive evaluation of the state-of-art of drug synergy prediction. Collectively this Challenge manuscript equips the scientific community with data and a methodological baseline for algorithm development, alongside a suite of computational methods to direct new experiments towards likely synergistic drug combinations.

The results of the AstraZeneca-Sanger Drug Combination Prediction DREAM Challenge have shed important light on the best practices and limitations in predicting drug synergy. By evaluating predictions from a large number of teams, we were able to discern important strategies for predicting drug synergy from molecular and chemical traits. As with most DREAM Challenges, we observed that the machine learning method itself has little impact on overall performance. Aggressive pre-filtering that incorporates clean sparse network data to consider feature relevance to drug targets and cancer was successfully used by top performers to limit model complexity and improve model generalizability. Despite the complexity of the problem, many teams reached the upper-bound of performance levels based on variability in experimental replicates. This was further confirmed when top-performing models were applied to an independent dataset, demonstrating robustness to assay variability, and context heterogeneity.

A comprehensive assessment of the predictive value of monotherapy was not completed in the Challenge format, in part due to initial miss-annotation of data. However, post-hoc analyses suggested it offered no significant improvement to well-performing models (Supplementary Fig. 12). Despite minimal predictivity from monotherapy itself as a feature, biomarkers associated with monotherapy resistance were observed to have predictive value for respective combinations. Looking forward, additional attention is also required for the one-fifth of combinations poorly predicted by all Challenge teams. The rationale differentiating these combinations is non-obvious but our data suggests, in part, some relationship to the complexity of network connectivity between drug targets and proximal biomarkers (Fig. 5c), perhaps a bias introduced by network-led dimension reduction techniques employed by well-performing models. Furthermore higher synergy scores were observed—in some combinations—when both drugs target downstream of a commonly interacting protein^22^. Collectively, these observations advocate for a more biologically rationalized approach to biomarker discovery, accounting for directionality and exclusivity of signaling and functional relationships between biomarkers and targets.

A notable absence from the Challenge was the use of mathematical, reaction- or logic-based mechanistic pathway modeling approaches^23–27^, likely due to the higher time and data input needed for model creation. The dynamic nature of mechanistic models may offer an advantage by enabling consideration of the heterogeneity that exists across even apparently ‘clonal’’ cell line populations^28^. The increasing availability of published pre-derived mechanistic models for many cancer relevant pathways may soon make such an approach more viable. Given the strong benefit seen from inclusion of prior-knowledge, and as text-based artificial intelligence technology matures, computational approaches, such as natural language processing (NLP) to harness knowledge from world literature may also become of significant benefit. Alternatively, more generic signatures of dynamic (e.g., transcriptional) output may first be used to identify a mechanistic rationale^28–31^ to which causative genetic or epigenetic events can then be inferred and aligned as predictive features^32,33^. A surprising result of our Challenge, however, suggested only modest improvement to prediction from inclusion of all data in SC1A compared to only genetics in SC1B.

To maximize potential for translation it is essential that modeling approaches reveal testable biological insight, particularly considering that this and prior Challenges show no predictive advantage to black box algorithms. As we discovered, however, it can be difficult to incentivize knowledge retrieval within a competition format that focuses on objective scoring of performance. Despite these limitations, we were able to extract important insights to biomarkers for certain drug combinations. Given the dominance of RTK and PI3K/AKT pathway targeting agents in the Challenge data, it was not surprising that these revealed some of our strongest combination-feature relationships. In multiple cases this aligned to a two-hit hypothesis targeting the activating driver with a downstream pathway component. These included synergies between EGFR and AKT inhibitors in the presence of activating *EGFR* mutations^34^, or AKT1/2 with pan-PI3K inhibitors in the presence of pathway activating mutations in *PIK3CA* or *PTEN*. In some cases the biomarker rationale for AKT inhibitor synergy with RTK or MAPK inhibition was less direct but indicative of crosstalk and feedback signaling previously reported^35^. Interestingly antagonism was observed in cell lines harboring activating mutations of *AR*^36–39^. Feedback signaling resulting from AKT inhibition has been seen to drive AR activity, which in turn can lead to the activation of the MAPK cascade^39,40^, attenuating respectively targeting drug activity.

The synergy observed between ADAM17 and PI3K/AKT pathway inhibitors may work through independent inhibition of multiple cancer hallmarks, or via a more direct mechanism whereby inhibition of ADAM17 driven proteolysis and shedding of RTKs^20^ stabilizes and increases signaling through PI3K/AKT^41,42^. Notably ADAM17 predominantly influences RTK’s other than EGFR/ERBB2^20^, and no benefit is seen in cells with mutations in these genes. ADAM17 inhibition, however, showed antagonism unique to combined PIK3CB/D selective inhibitors within the *PIK3CA* mutant setting. Reduced synergy may result from a lessened dependency on PI3K paralogues in the presence of constitutively activated PIK3CA, or reduced benefit from ADAM17 loss in the extreme luminal/epithelial physiology of *PIK3CA* mutants. The apparent antagonism, however, suggests feedback following PIK3CB/D inhibition enhances mutant *PIK3CA* expression/activity. Indeed PIK3CB inhibition has been shown to result in elevated expression and activity of PIK3CA^43^, and may also relieve the inhibitory effects of substrate competition or dimerization between PIK3CA and PIK3CB/D.

Many drug combinations effective in the clinic to date involve mechanistically distinct agents, often chemotherapies combined with an additional targeted therapy, for which benefit may arise from the independent effects of the drugs on different subpopulations^44^ rather than synergy. More recently, an increasing number of combinations include multiple targeted therapies^5^. Hence, identifying both molecularly synergistic and complementary drugs, and how these affect inter- and intra-patient heterogeneity remains an essential area of future research. Future Challenges should further address the question of how to optimize translation of preclinical results into the clinic^45^. Where this Challenge addressed prediction of synergy for combinations of known drugs, an ability to predict truly novel beneficial target combinations should also be explored. Furthermore, the space of therapeutic combinations should be extended to include >2 drugs, and covering targets in independent cell types, such as subclonal tumor cell populations or cells of the tumor microenvironment and immune system^3^. These approaches can be complemented by adaptive and sequential strategies reactive to monitoring of the patient tumor and physiology. Success in these areas will be dependent on the availability and access to large-scale data needed for model development and validation. Public-private partnerships—as exemplified by this Challenge and AstraZeneca’s generous sharing of data with the research community—will be critical to future efforts.

## Methods

### Drug combination screening

All cell lines were authenticated at AstraZeneca cell banking using DNA fingerprinting short-tandem repeat assays and each bank is confirmed to be free from mycoplasma. Cells ordered from the global cell bank are cultured for up to 20 passages. Cell suspensions are counted using a haemocytometer and cells are re-suspended in full growth medium containing Pen/Strep to a final density for different cell line densities and for different seeding densities into 384-well cell culture plate. A volume of cells as determined by cell count and dependent on cell type was added to each well of a Greiner 384-well plate using a Multidrop Combi liquid handler and then incubated at 37 °C and 5% CO_2_ overnight in a rotating incubator. After seeding, plates were shaken to distribute the cells more evenly at the bottom of the wells and left to stand on the bench for 1 h to allow even settling of cells.

Drug combinations were screened with four combinations per 384-well plate in a 6-by-6 range of concentration format. The first row and first column in the 6-by-6 matrix are monotherapies of each drug in the combination, while the top left corner is the untreated control. Drug combinations therefore were tested in a 5-by-5 layout with comprehensively rescreening the monotherapy and control for each experiment to minimize batch effects. Drug concentrations ran from the highest dose to the lowest dose. All plates were dosed with drugs solubilized in DMSO or PBS, or DMSO alone to provide comparable treatment and max control wells. After 5 days of incubation 5 µl of 2 µM Sytox Green working solution was added to each well of the 384-well plates (0.133 µM final concentration) and the plates incubated for 1 h at room temperature. After incubation plates were read by the Acumen laser scanner to detect the number of Sytox Green stained cells. The total fluorescent intensity across the well was then read and the number of dead cells calculated by dividing this total fluorescence by the fluorescence of a single cell. The plates were re-read on the Acumen to give a total cell count. A live cell count was then determined by subtracting the dead cell count from the total cell count.

### Quantifying combination synergy and antagonism

Synergy and antagonism were quantified in an automated way using freely available Combenefit software (v1.31)^11^. The approach implemented in Combenefit is based on quantifying synergy distribution by comparing a drugs combination experimental dose-response surface to a modeled reference based on individual drugs dose-response curves. Briefly, for each 6-by-6 matrix, monotherapy dose-responses were extracted and modeled as a sigmoidal curve via the Hill equation^46^. A reference dose-response surface is then generated by Combenefit based on the Loewe model of additive combinations and the single drug dose-response curves. The experimental combination dose–response surface is then compared by the software to the model-generated one, resulting in a synergy distribution in concentration space. This synergy distribution is finally further summarized by integrating the synergy distribution in logarithmic concentration space. The procedure resulted in a single score (the result of this integration) for each combination.

### In vivo response class definitions

Response data for 62 treatments across ~1000 PDX models were derived from Gao et al.^8^ Putative drug targets from the AZ-DREAM and Gao et al.^4,8^ dataset were utilized to identify overlap. As synergy scores were not available for the Gao et al.^4,8^ dataset, ‘Best Response’’ (complete response-CR, partial response-PR, stable disease-SD, progressive disease-PD) for each combination-PDX pair were extracted and compared with monotherapy ‘Best Response’’ of each drug in the combination on the same PDX model. This was represented numerically where CR = 4, PR = 3, SD = 2, and PD = 1. Synergy was assigned to a change of +2 or more, and Antagonism to a change of −2 or less. A change of +1, 0, or −1 was assigned Additive, considering an element of experimental variability. Cases where best response has been observed as a range over time (PR→→PD), the earliest response was considered as we hypothesize this to reflect in vitro response in a more realistic sense for comparison.

The percent tumor volume change class definitions are as following:

1. Synergistic efficacy: Combination treatment leads to better tumor regression than either monotherapy.
2. Synergistic non-efficacy: Combination response is better than either monotherapy but does not result in tumor regression.
3. Additivity: Combination response same as the better of either monotherapy responses.
4. Non-synergistic efficacy: Combination response weaker than the better of the monotherapy responses but results in tumor regression.
5. Antagonism: Combination response weaker than both monotherapies.

### In vitro response class definitions

Response scores defined by the Loewe synergy model were considered in ordered to define in vitro response classes. Synergism was defined as Loewe scores ≥20, Antagonism ≤ −20, and rest are classed as Additive.

### Molecular characterization

The 85 cell lines were molecularly characterized, including:

1. Mutations from whole exome sequencing with Illumina HiSeq 2000 Agilent SureSelect (EGAS00001000978)
2. Copy-number variants from Affymetrix SNP6.0 microarrays (EGAS00001000978)
3. Gene expression from Affymetrix Human Genome U219 array plates (E-MTAB-3610)
4. DNA methylation from Infinium HumanMethylation450 v1.2 BeadChip (GSE68379)

Mutations were called with CAVEMAN [https://github.com/cancerit/CaVEMan/]^47^ and PINDEL [http://gmt.genome.wustl.edu/packages/pindel/]^48^ as reported in ref. ^12^. Variants were provided without further filtering, including putative passenger mutations, germline variants, and potential cell line artefacts, which are in total 75,281 mutations in 85 cell lines.

Copy-number variants (CNVs) are called with the PICNIC [http://www.sanger.ac.uk/resources/software/picnic/]^49^ algorithm using the human genome build 38 as the reference. CNVs might be wild type, deletion, or amplification of certain segments in a chromosome. One or multiple genes can fall within such segments. We reported copy number for the major and minor allele on gene and segment level.

Gene expression was processed as described in ref. ^12^, including Robust Multi-array Average (RMA) normalization with the R-package ‘affy’’^50^. Gene expression for 83 cell lines across 17,419 genes (HGNC labels) was reported; no expression was available for MDA-MB-175-VII and NCI-H1437.

DNA methylation was reported for 82 cell lines the *beta* and *M* values^51^ for 287,450 probes; no methylation was available for the cell lines SW620, KMS-11, and MDA-MB-175-VII. In an additional processing step, CpG sites were compressed to CpG island with the definition from UCSC genome browser^52^, resulting in a total of 26,313 CpG island based on either *M* or *beta* values.

### Drug properties

The identity of all drugs was anonymized, but for all agents the putative targets are revealed. The gene names of the protein targets are listed with ‘*’’ denoting any character if the target is a protein family. Furthermore, for 58 drugs the Molecular weight, H-bond acceptors, H-bond donors, calculated octanol-water partition coefficient, Lipinski’s rule of 5, and their SMILES (Simplified Molecular Input Line Entry Specification) are provided. Drugs were grouped into pathways and biological processes manually according to their protein targets (Supplementary Data 1).

### Challenge organization

The Challenge consisted of two sub-challenges, each with multiple rounds: a leaderboard, validation, bonus, and collaborative round. SC1 had four leaderboard rounds that lasted 8, 6, 5, and 5 weeks, while SC2 had three leaderboard rounds that lasted 12, 7, and 5 weeks. Participants were given a leaderboard dataset to build a model and generate three prediction files per leaderboard round. Scores were returned to participants so that they can improve their model throughout these rounds for their one submission to the final round, which was scored against a held-out dataset. The final round lasted for 2 weeks, which was then followed by a 9 week bonus round and 10 week collaborative round.

### Challenge pharmacology data splits

In SC1, participants were asked to predict drug synergy of 167 combinations in the panel of 85 cell lines. The synergy data of each drug combination was partitioned into three sets: a training dataset (3/6–50%), a leaderboard set (1/6–16.7%), and validation set (2/6–33%) of treated cell lines. SC2 leveraged data for remaining 740 drug combinations not overlapping with those used in SC1, although data for some of the same drugs (in combination with different drugs), homologous drugs (i.e., same target, but different chemical structure), and cell lines were included. A leaderboard set (370 combinations) and a final validation set (370 combinations) were randomly split, which are mutually exclusive from each other, as well as from SC1.

### Primary scoring metric of Sub-Challenge 1

With synergy scores roughly normally distributed and and outliers truncated to −100 and 100 (Supplementary Fig. 2), Pearson’s correlation was employed as the base measure of prediction accuracy within each drug combination. The primary metric was then the average weighted Pearson correlation (*ρ*_w_) of the predicted vs. observed synergy scores across each individual drug combination, *i*. The weight for a given drug combination *i* was $\sqrt {n_i - 1}$where *n*_*i*_ is the number of cell lines treated with the drug combination. This resulted in the following primary metric for SC1A&B,

1 $$\rho _w = \frac{{\mathop {\sum }\nolimits_{i = 1}^N \sqrt {n_i - 1} \,\rho _i}}{{\mathop {\sum }\nolimits_{i = 1}^N \sqrt {n_i - 1} }},$$

where *N*` = 167 were the tested drug combinations.

### Tie-breaking scoring metric of Sub-Challenge 1

The tie-breaking metric was identical to the primary metric Eq. 1 except that it was applied to the subset of drug combinations that have at least one cell line with synergy score $S_{ci} \ge 20$in the held-out test set (*S*_*ci*_ = synergy score at cell line *c* and drug combination *i*). Neither the subset of drug combinations nor its size (*N* = 118) was revealed to participants prior to final evaluation.

### Primary scoring metric of Sub-Challenge 2

The primary metric was a sequential three-way ANOVA, which tested the separation of held-out synergy scores by predicted synergy (=1) and predicted non-synergy (=0). In the sequential three-way ANOVA (type 1), we controlled for systematic drug and cell line effects, and evaluated variance explained by a given team’s synergy predictions. We define the primary metric as

2 $${\mathrm{SA}} = - {\mathrm{sgn}} \times {\mathrm{log}_{{10}}}(p),$$

where sgn is the sign of the effect size (positive or negative separation by prediction), and *p* is the *P*-value (*F*-statistic) computed from the ANOVA distinguishing predicted synergy (=1) from predicted non-synergy (=0) across all experimentally measured synergy scores.

This three-way ANOVA score can be interpreted via linear regression where the intercept is set to 0.

3 $$y \sim \beta _1 \cdot {\mathrm{dc}} + \beta _2 \cdot {\mathrm{cl}} + \beta _3 \cdot x$$

Here, the response variable *y* is the observed synergy, which is normally distributed, and there are three predictive features: dc = drug combination, cl = cell line and a teams binary synergy predictions *x*. Then the primary metric, ${\mathrm{SA}} = - {\mathrm{log}}_{10}(p.{\mathrm{value}}[\beta _3])x{\mathrm{sign}}(\beta _3)$, measures the significance of a team’s predictions after controlling for variance associated with cell line and and drug combinations.

### Tie-breaking scoring metric of Sub-Challenge 2

As the tie-breaking metric, we used balanced accuracy (BAC) using discretized synergy scores $S_{ci} \ge 20$to evaluate the binary classifiers submitted in this sub-challenge. This metric evaluates both the sensitivity and specificity of the classifiers while taking into account the low proportion of synergistic cases to un-synergistic.

### Application of the Tie-Breaking Metric

In each sub-challenge, we estimated a Bayes Factor (BF) using a paired bootstrapped approach to determine whether a team’s score was statistically indistinguishable from another. In the event that a team’s scores were determined to be statistically equivalent, we then applied the tie-breaking metric. To estimate the BF, we sample with replacement from the *M* observations of the given sub-challenge and computing the primary metric (pm) for each team 1000 times. For a given team, *T*, *K*_*T*_ was computed by

4 $$K_T = \frac{{\mathop {\sum }\nolimits_{i = 1}^{1000} pm_{T,i} < pm_{best,i}}}{{\mathop {\sum }\nolimits_{i = 1}^{1000} pm_{T,i} \ge pm_{best,i}}}$$

Where $pm_{best,i}$is the bootstrapped primary metric at iteration *i* for the team with the highest primary metric (non-bootstrapped).

### Assessing performance of individual combinations

Combinations defined as poorly predicted had an average predicted vs. observed Pearson correlation across teams in the range seen with a random predictor (SC1 Primary metric = −0.25 and 0.25). In contrast, well predicted combinations had an average Pearson correlation across teams of above 0.5.

### Independent validation on O’Neil et al. Merck screen

In order to assess the utility of features and the predictability of the learning algorithms in new contexts, we provided the participants an independent large-scale oncology combination screen published recently^4^. The O'Neil et al.^4^ dataset consists of 22,737 experimental endpoints covering 583 doublet combinations across 39 diverse cancer cell lines. Thirty-eight experimental drugs and approved drugs were included in this combination screen using a 4-by-4 dosing regimen. Raw cell viability measures for each combination experiment were processed through Combenefit^11^ to generate synergy scores as per the Challenge dataset. While there are 6 approved drugs, 49 targets, and 10 cell lines in common between the Challenge and O'Neil et al.^4^ datasets, the total number of exact experiments (Drug A–Drug B–Cell line) overlapping is below 100, giving the participants a highly independent validation set for their prediction algorithms. This information was provided to best performing teams in the Challenge, along with a dictionary of curated chemical structures and putative targets for each. Prediction models were trained on the released Challenge dataset and made synergy score predictions on the O'Neil et al. dataset. Metrics for SC1 and SC2 were used to assess prediction performance.

### Individual prediction models

Full description and implementation of models used by teams in the final submission to DREAM can be downloaded from:

Synapse.org/AstraZeneca_Sanger_Drug_Combination_Challenge_Leaderboards [http://www.synapse.org/AstraZeneca_Sanger_Drug_Combination_Challenge_Leaderboards]. Top-performing prediction models in SC1 and SC2 made use of genetic features relating to the gene targets of the drugs. Feature selection from the models enabled nomination of putative biomarkers for drug combination synergy (Supplementary Methods).

### Ensemble models

SC2 participant models were aggregated using two types of ensemble models Spectral Meta-Learner (SML) and Random Aggregation. SML choses predictions from *n* methods to aggregate based on an estimation of BAC for each method without using actual labels^15,53^. Random Aggregation is the traditional way that people aggregate models by giving equal weight to each method. We randomly pick *n* methods (do this ten times) and for *n* methods we compute the average BAC and the error.

### Monotherapy biomarkers and synergy enrichment

Monotherapy markers are the mutational status of genes, either mutated or copy number altered, from the pan-cancer binary event matrix (BEM)^12^, which separate the monotherapy response into sensitive vs. non-response. The likelihood of separation was estimated with a Wilcoxon Rank Sum test (Supplementary Methods). From most significant monotherapy marker to lowest in 0.1 steps of –log10(*P*-value), we accumulative evaluated the percentage of synergistic combinations (synergy score ≥20) with at least one monotherapy marker. This analysis was bootstrapped five times with 80% of the pharmacology data (Supplementary Methods).

### Synergy biomarkers

A short list of putative synergy biomarkers were derived from the five highest ranked features of well predicted drug combinations (Pearson *r* > 0.5) from the two best performers NAD and DMIS. Features were ranked based on their feature weight or importance for given well predicting model. This gene-to-combination short list, was filtered for associations predicted by both teams, or genes biological related to the drug target defined as either the gene being the target itself, a short distance to it in OmniPath signaling network (two molecules up- or downstream) or GO term similarity^54^ larger than 0.7. This resulted in a list of 47 gene-to-combination associations that we further studied. A gene within this list is considered mutant if it was deleted, amplified (>7 copies) or mutated in any sense, resulting in an extended BEM^12^. We calculated the *P*-value for each suggested association with an ANOVA correcting for tissue of origin and multiple hypothesis testing via Benjamini Hochberg. The effect sizes is the mean difference in synergy score between mutant and wild-type cell lines.

For external validation of those putative biomarkers of synergy, we focused on drug combinations in O’Neil et al.^4^, ALMANAC^13^, and additional experimental data from AstraZeneca (Supplementary Data 3). We validated biomarkers in two different contexts, (i) for cell lines overlapping with AZ-DREAM, considered as biological replicates, and (ii) cells non-overlapping for predictions on independent cell lines.

Literature evidence for the shortlisted combination-biomarker associations was identified through PubMed search. The aim was to identify published evidence of (i) the combination-biomarker association, (ii) the combination but not the specific biomarker, and (iii) either one of the targets and the biomarker association. The publications were further categorized into in vitro, in vivo, and preclinical studies. Publications that discuss the specific combination-biomarker association have been highlighted in red (Supplementary Data 3). In summary, synergy biomarker were derived from best performer models, and highlighted based external validation, as well as literature support.

### Reporting summary

Further information on research design is available in the Nature Research Reporting Summary linked to this article.

## Supplementary information

Supplemenatary Information

Description of Additional Supplementary Files

Supplementary Data 1

Supplementary Data 2

Supplementary Data 3

Reporting Summary

## Data Availability

The data used for this study are available from the Genomics of Drug Sensitivity in Cancer (GDSC [http://www.cancerrxgene.org/downloads/]) repository^12^, the Catalog of Somatic Mutations in Cancer (COSMIC[http://cancer.sanger.ac.uk/cell_lines]) database^55^, the European Genome-phenome Archive (EGA) EGAS00001000978, Gene Expression Omnibus (GEO) GSE68379, ArrayExpress E-MTAB-3610, Synapse database synapse.org/DrugCombinationChallenge[https://www.synapse.org/DrugCombinationChallenge] and the AstraZeneca Open Innovation Portal openinnovation.astrazeneca.com/data-library.html[https://openinnovation.astrazeneca.com/data-library.html].

## Code availability

Full description and source code of all participants in this Challenge can be downloaded from synapse.org/DrugCombinationChallenge[https://www.synapse.org/DrugCombinationChallenge].
