# Supplementary material for: Community assessment to advance computational prediction of cancer drug combinations in a pharmacogenomic screen
Source: Nat Commun. 2019 Jun 17;10:2674. doi: 10.1038/s41467-019-09799-2 (PMC6572829; doi:10.1038/s41467-019-09799-2)
Supplement: Supplementary file 1 — Supplemenatary Information [file 41467_2019_9799_MOESM1_ESM.docx]

**Community assessment to advance computational prediction of cancer drug combinations in a pharmacogenomic screen**

Menden, Wang, Mason, Szalai, Bulusu, Guan et al. 2019

#

# Supplementary Methods

## Quality assessment of pharmacology data

The Quality Assessment (QA) flag is an output from Combenefit, which was used for the synergy score and monotherapy curve fitting (see Online Methods). Scores are defined as the following:

| **Flag** | **Meaning** |
| --- | --- |
| 0 | No data was found in a combination folder or NaN was found in a combination file. |
| -1 | At least one of the measured drug effects was above 125% of starting cell count. This is unlikely to be genuine and a major experimental issue is suspected. |
| -2 | No flag '-1' but measured effects below -10% were found. By definition, effects should always be positive because cell viability is being measured. Very small negative values are sometime encountered due to quantification problems at high concentrations. These were tolerated up to -10% below which major issues were suspected. |
| -3 | No flags '-1' or '-2' but combination dose-response showed very strong fluctuations. The combination dose-response was smoothed and compared to the original non-smoothed version. If differences above 25% were found the experiment was flagged as measurements likely to be unreliable. |
| 1 | None of the previous problems were encountered. Data is supposed to be ok. |

All experiments were observed to have some level of synergy or antagonism and had non-zero synergy scores. Most of these were due to random variation in the experiments and had synergy within +/-1, and only 404 experiments with low variability non-zero experiments due to quality issues in the assay and were flagged accordingly (Fig. S1B). Only high quality data (QA=1) were included in the testset, while experiments also with low quality were made available for training.

### **Drug combination synergy is variable across cells, but reproducible across replicates**

367 had a replicate experiment where the same drug combination, cell line and concentration ranges tested (Fig. S2A). The Spearman correlation between synergy scores across the replicates was 0.56, which is comparable to the correlation of 0.63 from the 315 replicate combinations screening experiments (Fig. S2B) completed by O’Neil *et al*[*^1^*](https://paperpile.com/c/hsaAvY/MlDer). We observed that in all instances where the synergy and antagonism were not measured by both replicates, the quality of one or both replicates had been flagged as low (see above QA flags). Notably, the variance in synergy scores in AZ-DREAM (Fig. S2C) was larger than the variance the dataset from O’Neil et al (Fig. S2D).

### **Monotherapy biomarkers and synergy enrichment**
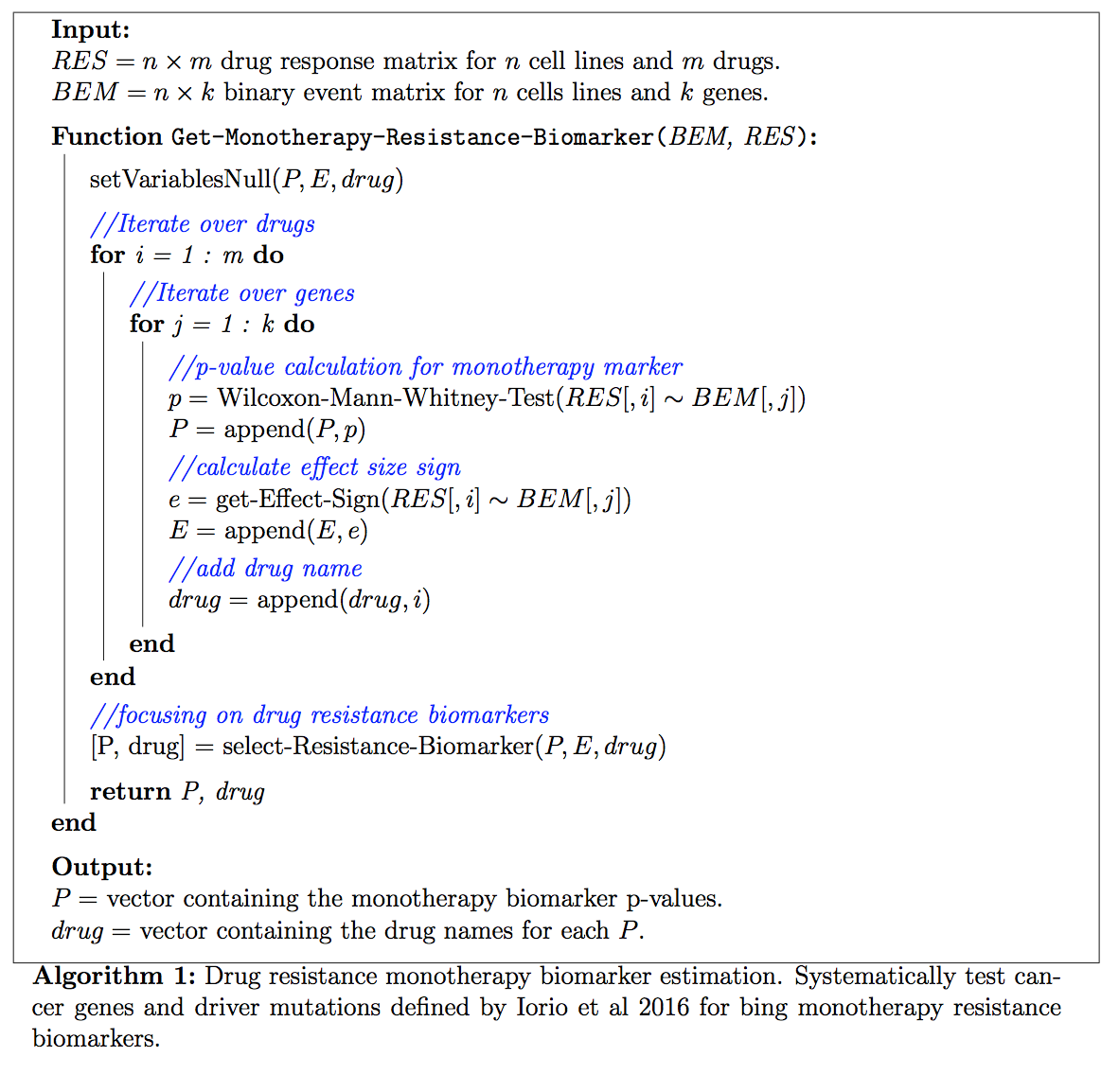

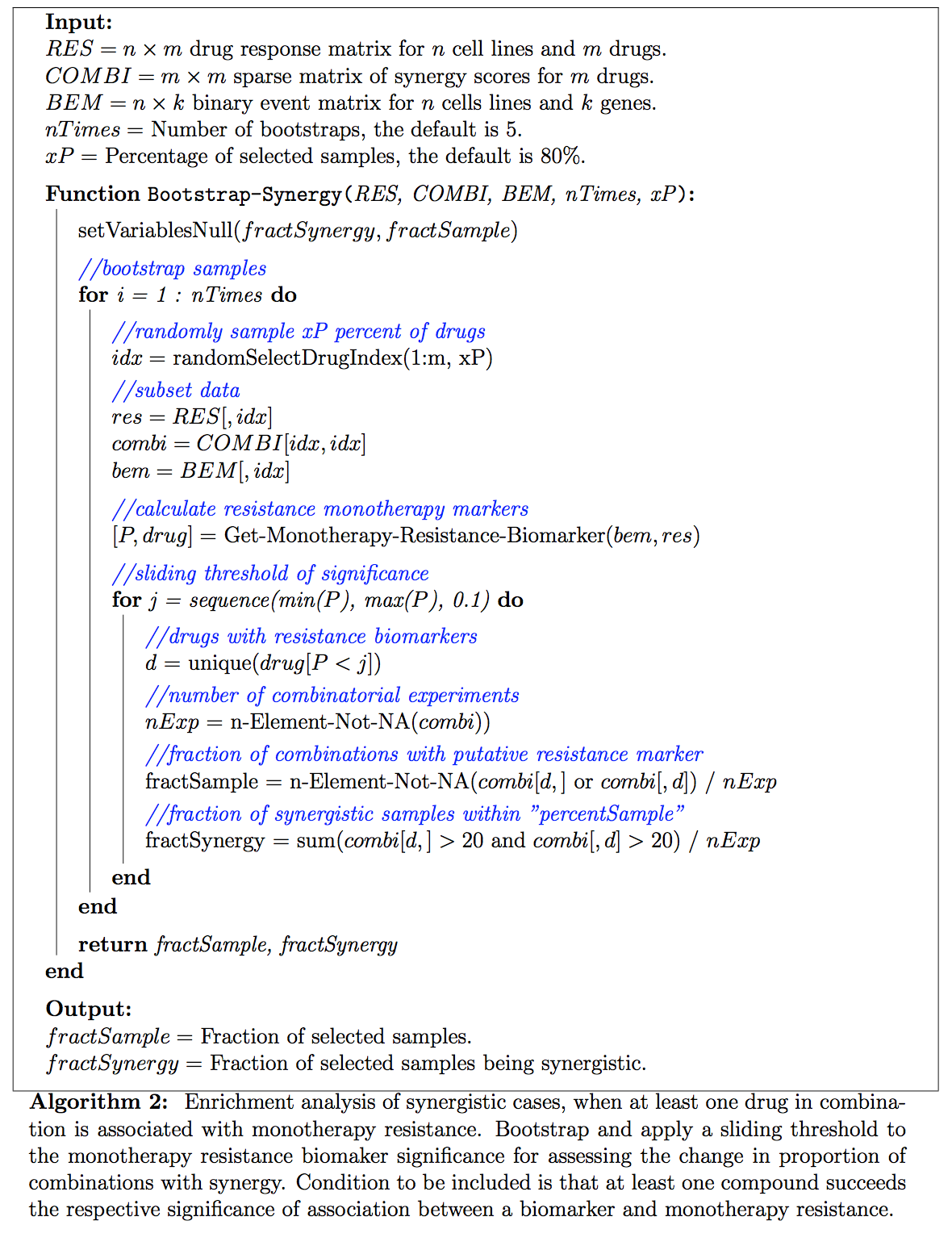


##

## Best ranked teams’ Methods:

Best ranked teams’ methods are detailed below, for teams who performed consistently well across sub-challenge 1 (SC1), sub-challenge 2 (SC2) and sub-challenge 3 (SC3), or outstanding well for at least one sub-challenge.

### Yuanfang Guan

This method begins by limiting the feature space to those features (expression, copy number variation (CNV), methylation, mutations) mapping to genes that are putative targets of any drug in a given sub-challenge. If a drug is less specific and has multiple targets, all of them are included.

For each drug combination a separate classifier is built for predicting synergy. Each drug combination classifier is made of several random forests, and each using as distinct data type. For example, in SC1A three classifiers are created per drug combination, and with their predictions are averaged (table 1). This use of one classifier for each data type was motivated to improve the stability of the predictions, in the case that one of the feature sets contains outliers.

| SC1A Classifier | Data type |
| --- | --- |
| 1 | Mono-therapy data of Drug A and B |
| 2 | Drug A, B count data |
| 3 | All DC’s data with either Drug A or B |
| SC1B Classifier | Data type |
| 1 | CNV, mutation (and mean values for both), count on drug A, |
| 2 | CNV, mutation, count (and mean) on drug B |
| 3. | Normalized CNV, mutation (and mean), count on drug A and B |
| SC2 Classifier | Data type |
| 1 | Drug A, B, count, |
| 2 | Normalized All DC’s data with either Drug A and B |
| 3 | Drug space as a simple parsing of original table provided in the Challenge |
| 4 | Mono-therapy data of Drug A and B |
| Table 1 | |

The choice of classifier construct, randomForest, compared to using an SVM, regression, or boosting, was chosen for expedience, not any perceived advantage in accuracy, though the randomForest does facilitate prediction in a nonlinear context [^2^](https://paperpile.com/c/hsaAvY/obeGw). Instead, greater prediction accuracy is achieved by creating new features that consist of scaling the features provided by AZ-Dream with a posterior probability from a predefined functional network [^3^](https://paperpile.com/c/hsaAvY/NzTQ2).

*Network Based Feature Scaling:*

Feature scaling is motivated by the observation that when we use the original features as input, we found the prediction values are similar for the same cell line, regardless of drug perturbations. This is due to the fact the genomic and expression data are static for a given cell line across all drug combinations effectively leaving just three parameters for modeling drug synergy (drug A, drug B and cell line). Scaling cell line features by the functional network of a drug target creates a dynamic parameter space for each cell line that allows for better modeling synergy.

For gene expression, methylation, and copy number variation data, features are adjusted based on their probability of a functional relationship with drug target genes. Feature *x_i_*, associated with gene *i*, is scaled to x’*_i_* by:

$\{if g_{i}\in{(DT}_{A}, {DT}_{B}),{x'}_{i}=0 {else, x'}_{i}=x_{i}\times(1-max (e_{ij}) , \forall g_{j}\in{(DT}_{A}, {DT}_{B}) \}$ (1)

where *DT_A_* and *DT_B_* are the genes targeted by drug *A* and *B* respectively, and *e_i_*_j_ is edge between genes *i* and *j* in the predefined functional relationship network [^3^](https://paperpile.com/c/hsaAvY/NzTQ2).

For gene mutations, features are modified using the edge directly. Mutation feature *x_i_*, associated with gene *i*, is changed to *x’_i_*:

$\{if g_{i}\in{(DT}_{A}, {DT}_{B}),{x'}_{i}=x_{i} else, {x'}_{i}=x_{i}\times max (e_{ij}) , \forall g_{j}\in{(DT}_{A}, {DT}_{B}) \}$ (2)

The purpose of generating this new series of features is to simulate the effective values of expression, methylation, CNV and mutations post treatment. After scaling (Supplementary Equation 1), predictive features are different for each cell line across different drug-combinations. We reduce the effective values of drug target in expression, methylation and CNV to zero, and reduce the values of other genes according to their connections to the drug target. For mutations, we assumed that the effects of drugs are equivalent to adding in new mutations to the system, with the effect values of drug targets being 1 (similar to mutated genes), while the other genes are increased in values according to their connections to the drug target (Supplementary Equation 2).

A biological interpretation of this approach is that scaling cell line features with the functional network’s edges, allows the RandomForest to model a drug’s propagation through a targeted pathway cascade.

The weighting of different set of features was primarily done by cross-validation. However, we found that a single set of genomic features is often sufficient to achieve a similar performance as the entire set.

### Mikhail Zaslavskiy

The model is an ensemble of three individual models trained exclusively on categorical features describing drug and cell line identities and one model trained on categorical identity features plus corresponding drug MonoTherapy results. There are two main ideas at the core of the proposed model. First, it is very easy to overfit when dealing with biological data, so the key factor is a proper design of the cross-validation scheme which covers not only meta parameter estimation but also model selection steps. In addition to avoiding overfitting pitfalls, the cross-validation design is important to address precisely the sub-challenge questions defined by corresponding training/test splits. Second, a rich sampling of the experimental space provides an excellent support for a competitive model even without additional features describing biological entities under consideration. When we have enough data on drug/drug and drug/cell line combinations (like in SC1A and SC1B) we can derive the information on drug and cell line similarities without using additional features. Of course, it is impossible to know in advance if the experimental results alone are enough to reach the maximum performance, so the model building process is to start with the set of baseline features (drug and cell line ids) and then add step-by-step more complex features (MonoTherapy results, drug features, cell line mutations, copy number variations et c.) verifying at each step if the addition of new features lead to an improvement of the cross-validation score.

The three individual models used to predict drug synergy from durg/cell line identities are a gradient boosting tree model (xgboost package) and an svm model (e1071 package) trained on original identity features represented by a binary matrix, and an elastic net model (R/glmnet package) model trained on average scores of drug-cell line combinations with the same drug combination and a different cell line, average scores of drug-cell line combinations with the same cell line and one drug in common, average scores of drug-cell line combinations with the same cell line and no common drug. The fourth model is another gradient boosting tree model trained on drug MonoTherapy results and counts of categorical identity features. All four models were trained using 5-fold cross-validation, the final score was computed as a simple average of the individual models.

### North Atlantic DREAM (NAD)

North Atlantic Dream team's solutions used different tree based models (Random Forest Regression and Extreme Gradient Boosting Trees, XGBoost [^4^](https://paperpile.com/c/hsaAvY/oXd3k)) to incorporate the presumed important interactions between cellular (mutations, copy number alterations etc.) and drug specific (drug targets, affected pathways etc.) features. For better representation of the similarities between cell lines and drug combinations, new sets of features were engineered using prior knowledge and also the monotherapy data.

The monotherapy data (IC50, Einf, Hill slope) for a drug combination was dependent on the ordering of the drugs in the combination, which seemed to be hard to represent in the machine learning model. To overcome this problem, North Atlantic Dream's model used monotherapy features that are independent of the ordering (such as min/max/absolute difference etc. type features from the original data). Also the expected volume under the dose-response surface (in case of additivity) was calculated using the original Loewe model [^5^](https://paperpile.com/c/hsaAvY/OnM8x).

As the number of training examples for a given drug combination was relatively low (about a dozen for most combinations), it was crucial to find similarities between combinations beyond the trivial ones (same drug / same target). North Atlantic Dream created different feature sets based on GO (Gene Ontology)[^6^](https://paperpile.com/c/hsaAvY/w9zeh) / KEGG (Kyoto Encyclopedia of Genes and Genomes) Pathways [^7^](https://paperpile.com/c/hsaAvY/KqWNw)and a directed signaling network [^8^](https://paperpile.com/c/hsaAvY/wzE3N). For Gene Ontology based features a set of “cancer related” GO terms were selected (based on [^9^](https://paperpile.com/c/hsaAvY/IQMKT)), and for each drug a GO vector was created based on the association of GO terms with the target of the drug. For KEGG Pathway based features, KEGG Pathways containing the target genes were selected, and for each drug a KEGG vector was created, giving 1 values for pathways containing the target of drug, 0 otherwise. The GO/KEGG features for drug combinations were the sum of the two drug vectors of the combination. Based on the directed signaling network, for each drug combination the “similar” drug combinations were selected. Two drug combinations were defined similar, if the two targets of combination A are direct upward from the two targets of combination B. Based on this rule a similarity vector was created for each combination. To create these GO/KEGG/signaling networks based features, in case of “DNA targeting drugs” (i.e. chemotherapy drugs), the respective DNA damage response molecule was used as indirect target (based on Woods & Turchi, 2013 [^10^](https://paperpile.com/c/hsaAvY/EY1CY)).

For cellular features mutations, copy number variations and gene expression were used. The main problem with cellular features was their large number. To overcome this problem, North Atlantic Dream team used a pre-assembled gene list (including target genes, known oncogenes and tumor suppressors, genes related to drug monotherapy resistance etc. [^11^](https://paperpile.com/c/hsaAvY/FsIHH) [^12^](https://paperpile.com/c/hsaAvY/NXJwB) [^13^](https://paperpile.com/c/hsaAvY/qXKmo)). Genes with mutations / copy number alterations were selected from this gene list. The low number of training examples for a given drug combination made it hard to use traditional feature selection/reduction methods. However, based on the drug similarities defined above, it was possible to select molecular features associated with the observed synergy scores for a given, similar set of drug combinations. During the original Challenge gene expression features were selected (from the expression of target genes and their direct neighbors in signaling network) using this method by Randomized Lasso Feature selection. In the later, collaborative phase of the Challenge, a similar method was used for mutation and copy number variation features.

For the final prediction different XGBoost models were created using subsets of the above defined features and/or different model parameters. The final submitted predictions were the ensembles of these models, either as simple averages or using hillclimbing [^14^](https://paperpile.com/c/hsaAvY/Os15s)on out-of-fold predictions. For the various tasks through the Challenge R, Python, SAS, and JMP were used.

**NAD feature layer importance:**

NAD’s Random Forest Regression model was trained using different pairs of cell line and drug combination specific features. The tested cell line features included cell line label, mutations (pre-filtered for 469 cancer related gene) and CNV (pre-filtered for 292 gene). Combination related features tested here were drug label, drug target, Gene Ontology and KEGG pathway based features (feature size: 407 and 140, respectively) and signalling network based features (601). Baseline model used cell line and drug label as features, while in the other models the respective feature was either swapped with the corresponding baseline feature (e.g.: in *CNV model* CNV and drug label, in *target model* cell line label and drug target was used as features) or added to the features of baseline model (e,g. in *+target model* cell line label, drug label and drug target was used). Ensemble model in this case is the simple average of the prediction of these models. With all the used models 10 random cross-validation was performed, and the mean weighted Pearson correlation was calculated for each cross-validation run. For the cross-validations all the training and leaderboard data of the Challenge was used, and the size of the cross-validation set for each combination resembled the size of the test set of the Challenge.

**NAD biomarker selection:**

NAD ranked their biomarkers for each drug combination based on the number of times the feature was used for predicting the given combination in the Random Forest Regressor models. For each combination a separate Random Forest model was built (using mutation, CNV, drug target and KEGG features) where all the Challenge training and leaderboard data was used without the data of the actual combination. With this model the left out combination was predicted for all of the cell lines. The number of times a given cellular feature (mutation or CNV) was used (based on the internal structure of the Random Forest trees) was recorded for each combination - feature pair. For each drug combination - feature pair Mann-Whitney U test was performed to calculate the probability that the given feature is used more often for the given combination than other features, and that the given feature is used more often for the given combination than for other combinations. The final score of the feature for the combination was the product of these two probabilities. For each combination the used cellular features (mutation and CNV) was sorted decreasing order, and the top 5 features was used for further analysis.

### DMIS

Support Vector Regression (SVR) were used as prediction model. The main difficulty was the high dimensionality problem of our feature space. To address this, novel literature-based approach was used. 200 genes were identified that most frequently occur in the context of cancer in the literature, and used only the mutations in those 200 genes as the features. To perform this gene selection task, the Biomedical Entity Search Tool (BEST) (<http://best.korea.ac.kr>)[^15^](https://paperpile.com/c/hsaAvY/WYk8C) was applied. BEST finds an entity relevant to a query based on the number of co-occurrences between the query terms and the entity in the PubMed corpus, the authority of journals, the recency of articles, and the term frequency inverse document frequency (TF-IDF) weighting. BEST was queried by using the query term “cancer” and the top 200 cancer-related genes were collected. For CNV features, cBioPortal was used to collect 13 gene sets of cancer-related pathways. During the creation of various types of features, it was essential to identify the best combinations of feature groups. However, testing all possible combinations of feature groups would require a considerable amount of computing resources. To address this problem, a high performance computing pipeline using HTCondor was constructed. HTCondor is an open-source computing framework for coarse-grained distributed parallelization of computationally intensive tasks. We ran our pipeline using 1,764 cores from Amazon Web Service, and selected the best combination of the feature groups. Through this process, the following features for sub-challenge 1B were selected: 118 drug IDs, 99 drug targets, 94 CNVs, 241 mutations, and maximum concentrations of the dosages for each sample.

The SVR model showed a good performance on AZ dataset. However, because the original model represents target and mutation features as sparse binary vectors, it is not appropriate to apply to O'Neil et al dataset with unseen cell lines and drugs. For translatability, a dense vector was created to capture and generalize characteristics of cell lines and drugs, and constructed a deep learning model which could utilize these vectors as input.

For the post-hoc analysis, an additional deep learning model was generated, which was composed of 6 layers including a preprocessing layer as the first layer. The second layer had 4 modules and the first module gets mutation features, and the second module gets target feature. These two modules embed sparse feature vectors as dense vectors, and generate a single vector using a convolutional neural network. Pre-trained mutation vectors were used to leverage mutation information from TCGA and Mikolov’s Word2Vec algorithm[^16^](https://paperpile.com/c/hsaAvY/Z1k8z) for mutation embedding. In addition, Asgari’s public protein dense vector[^17^](https://paperpile.com/c/hsaAvY/kjoJa) for target embedding was used. The third module gets drug or monotherapy-related features, and the last module gets cell line-related features. Each module generates a single vector and the vectors are concatenated and are inputted into the next layer. The rest of layers are fully-connected layer. The output layer generates a single value, which is the predicted synergy score.

**DMIS feature layer importance:**

In the main Challenge, the Support Vector Regression (SVR) was used as machine learning model. For extracting feature layer importance, the accuracy of the model was estimated after randomly permuting the values of the feature. How much the permutation decreases the primary score of the SVR model is an estimate of feature layer importance.

To assess the importance of particular combinations of features we created 10 random partitions of the training dataset. To create one partition we randomly selected 1089 samples from the training set to mache that the size and the distribution of the final test set withheld by the organizers (i.e., same #samples for the same combo as in the final test set). The remaining data is used as training in this scenario. We repeated this 10 times to get 10 datasets.

In each partition we assessed the accuracy of the predictor after removing individual features and pairs of features from the 8 feature set. After removal of a given feature or feature pair, optimal C and Gamma were determined using the remaining features.

We computed the mean decrease in accuracy after for each removed feature(s) across the 10 dataset partitions where the decrease in accuracy is defined as the [primary metric *with* given feature] - [primary metric *without* given feature].

**DMIS biomarker selection:**

In order to get biomarker indications, important features were extracted for each drug combination individually. Therefore, samples were grouped by drug combination and for each combination and a random forest model was build. The method called “mean decrease impurity” was applied to obtain feature rankings. Random forest consists of multiple decision trees. Each node in a tree correspond to a feature and the same feature can appear in multiple trees. Each node in a tree splits the samples so that similar synergy scores group together. At each node, we can compute how much the split reduces the variance in the sample by taking the difference between the variance-before-split and the variance-after-split (this can be measured by weighted average of the two split groups). Finally, features were ranked based on their mean variance reduction, i.e., mean decrease impurity. The number of trees was limited to 200 and each tree randomly selected up to 204 features (a third of total 612 features available).

##

##

## Supplementary Figures


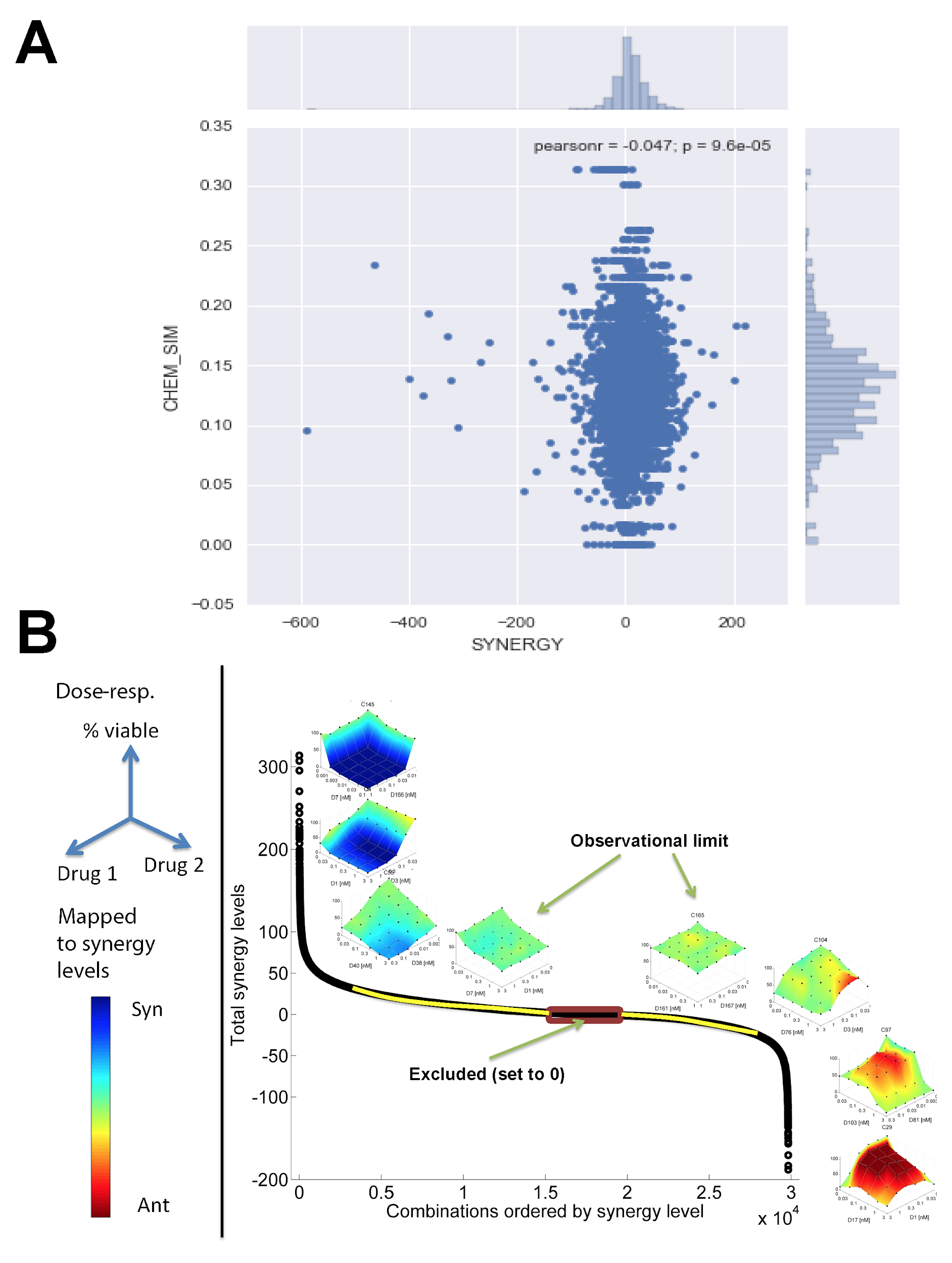


**Supplementary Figure 1**: Distribution of synergy scores across all combinations. (A) Chemical similarity of compounds in each combination is plotted against their synergy scores. (B) Chemical similar synergy scores of combinations are ordered from lowest to highest. 3D synergy heatmaps show additional cells killed (Syn) or not killed (Ant) beyond the additive effect of the two drugs at each dose. Two examples of combinations with total synergy scores of +/-20 show the limit at which synergy and antagonism can be visually confirmed. Experiments where non-zero total synergy was due to random variation were set to zero.


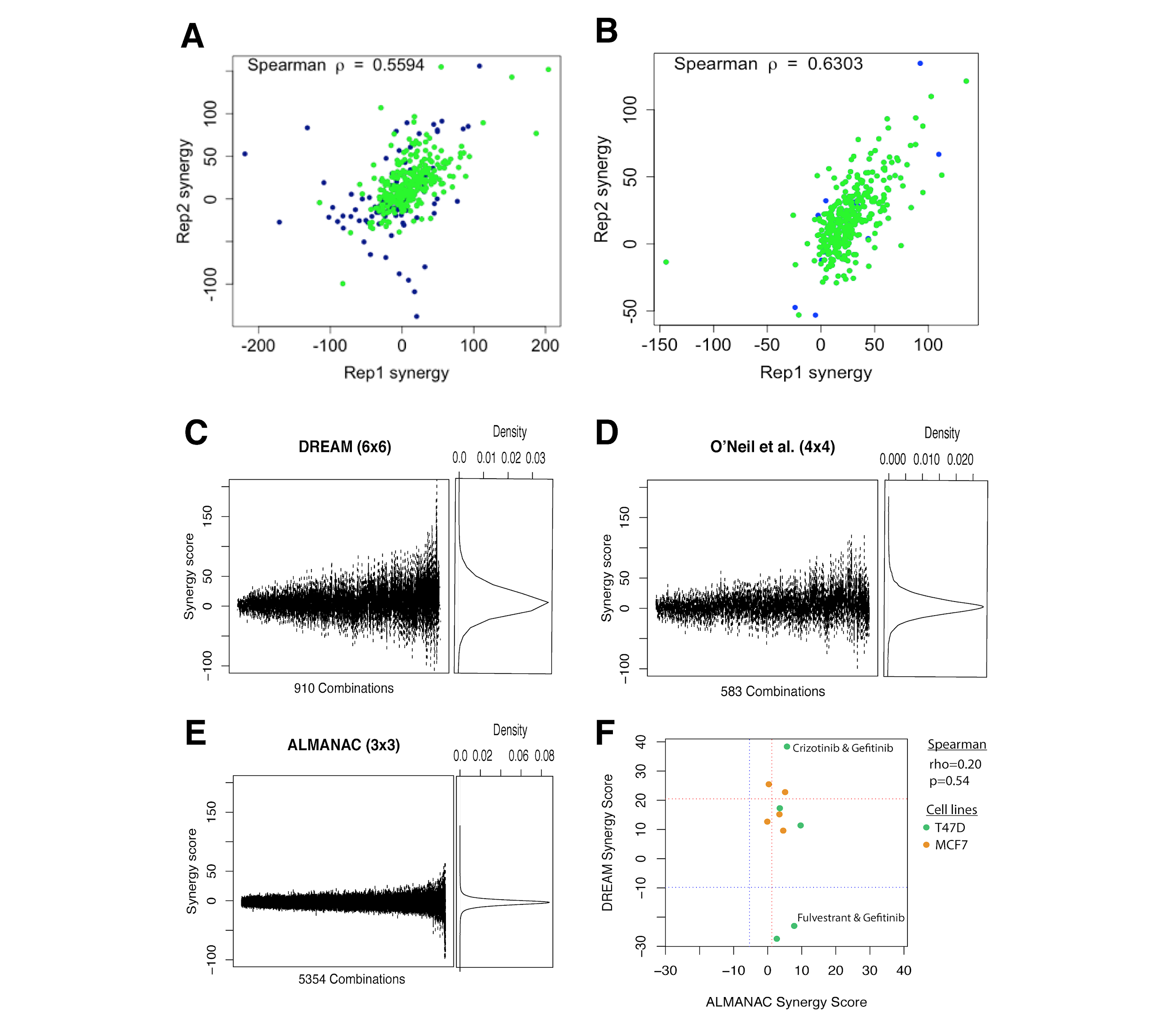


**Supplementary Figure 2**: Reproducible of synergy scores. Shows correlation of drug combinations replicates from (A) DREAM Challenge and (B) external combination screen (O’Neil et al. 2016). In green are high quality data points, while in blue are points not passing the QC from CombeneFit (QC score=1). (C, D, E) Distribution of synergy scores across combinations screened for the DREAM Challenge and independent sets of combinations screened by O’Neil et al and ALMANAC. (F) Comparison of synergy scores between DREAM and ALMANAC for the same combinations and cell lines tested. Dotted lines indicate the first (blue) and third (red) quartiles of synergy values for each dataset.


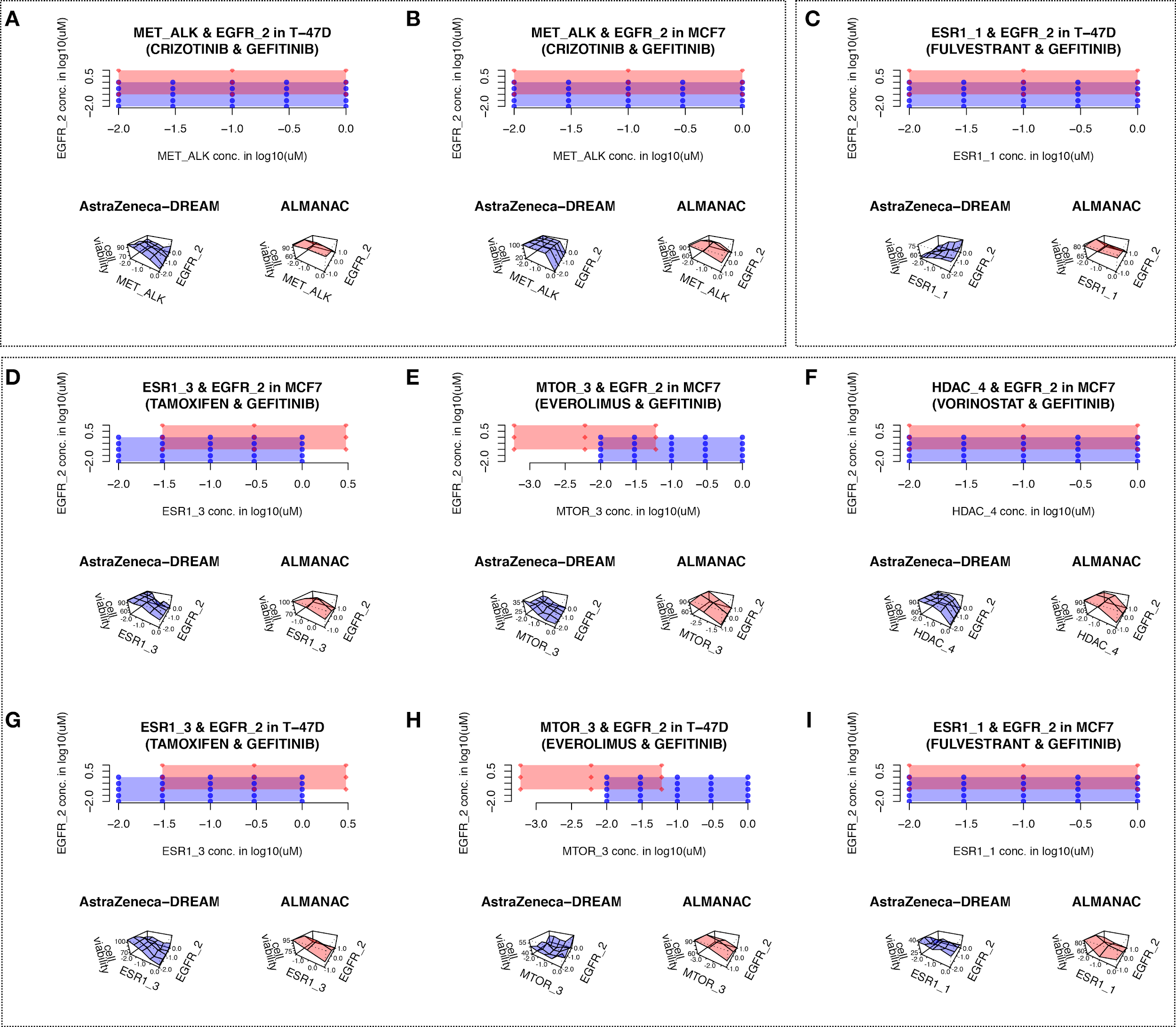


**Supplementary Figure 3:** Drug concentration range overlap between ALMANAC and AZ-DREAM for 9 experiments testing the same drug combination in the same cell line, and their experimental cell viability. Each panel is split into 3 parts: top part shows the overlapping concentration range from ALMANAC and AZ-DREAM, where red and blue dots are the titration points of ALMANAC and AZ-DREAM, respectively. Notably, in 38 instances the titration points are directly overlapping. The bottom left and bottom right part are the experimental cell viabilities across the drug combinations in log10 uM.


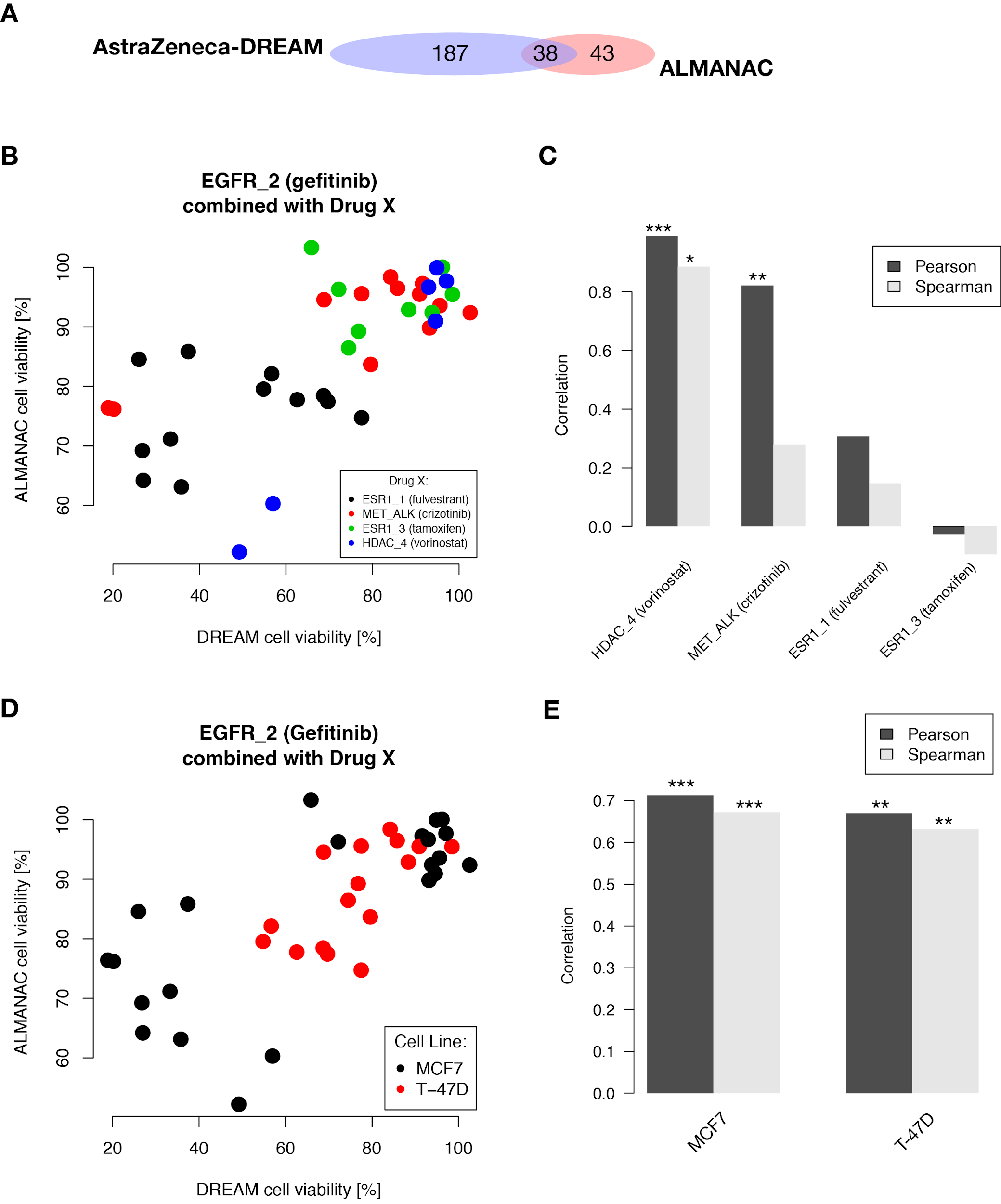


**Supplementary Figure 4:** Analysis of experimental points with exact identical combination concentration in ALMANAC and DREAM. (A) shows the overlap of 38 experimental data points, which have identical concentrations. (B) plots the estimated cell viability for ALMANAC and AZ-DREAM and color code the source experiment. (D) same plot as (B), but color codes for drug combination which is statistically quantified in (D). (C) is focusing on the cell lines, and (E) quantifies the observed correlations. Significant correlations are flagged as the following: *P<0.05, **P<0.01, and ***P<0.001.


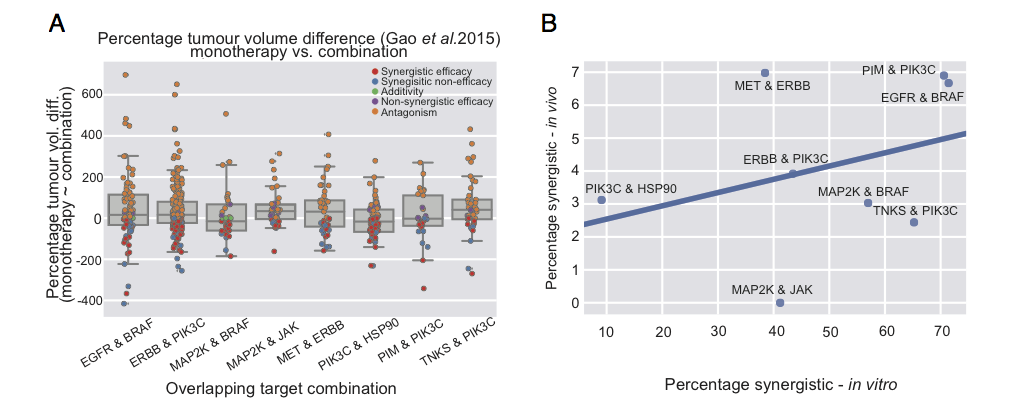


**Supplementary Figure** **5**: A) Summary of responses (% tumour volume change) for overlapping combinations between *in vitro* (AZ-DREAM) and *in vivo* (Gao *et al.*) datasets. (B) Correlation between % synergistic samples across *in vitro* and *in vivo* (regression line indicated).


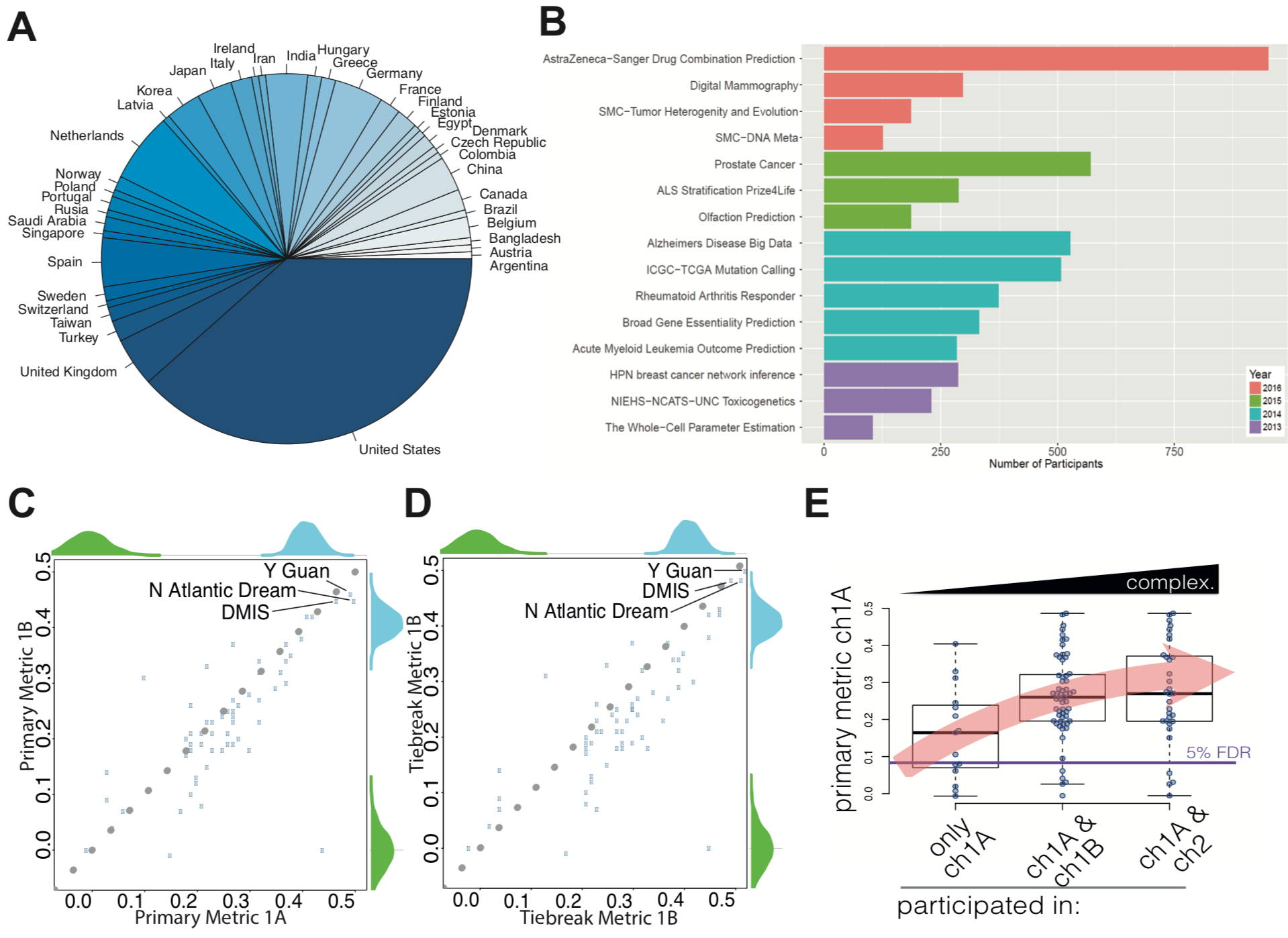


**Supplementary Figure** **6**: Participation in the Combinations Prediction DREAM Challenge. (A) Nearly 800 participants were located across five continents. (B) Comparison of participants across different DREAM Challenges. (C) Performances of sub-challenge 1A plotted against 1B based on the primary metric, average weighted Pearson correlation. (D) Performances of sub-challenge 1A plotted against 1B based on the tie-break metric, average weighted Pearson correlation of combinations with cases of synergy > 20. Dotted grey line shows 1:1 relationship between 1A and 1B performance. (E) Performance of participants in sub-challenge 1A grouped by whether they participated in one or more sub-challenges.


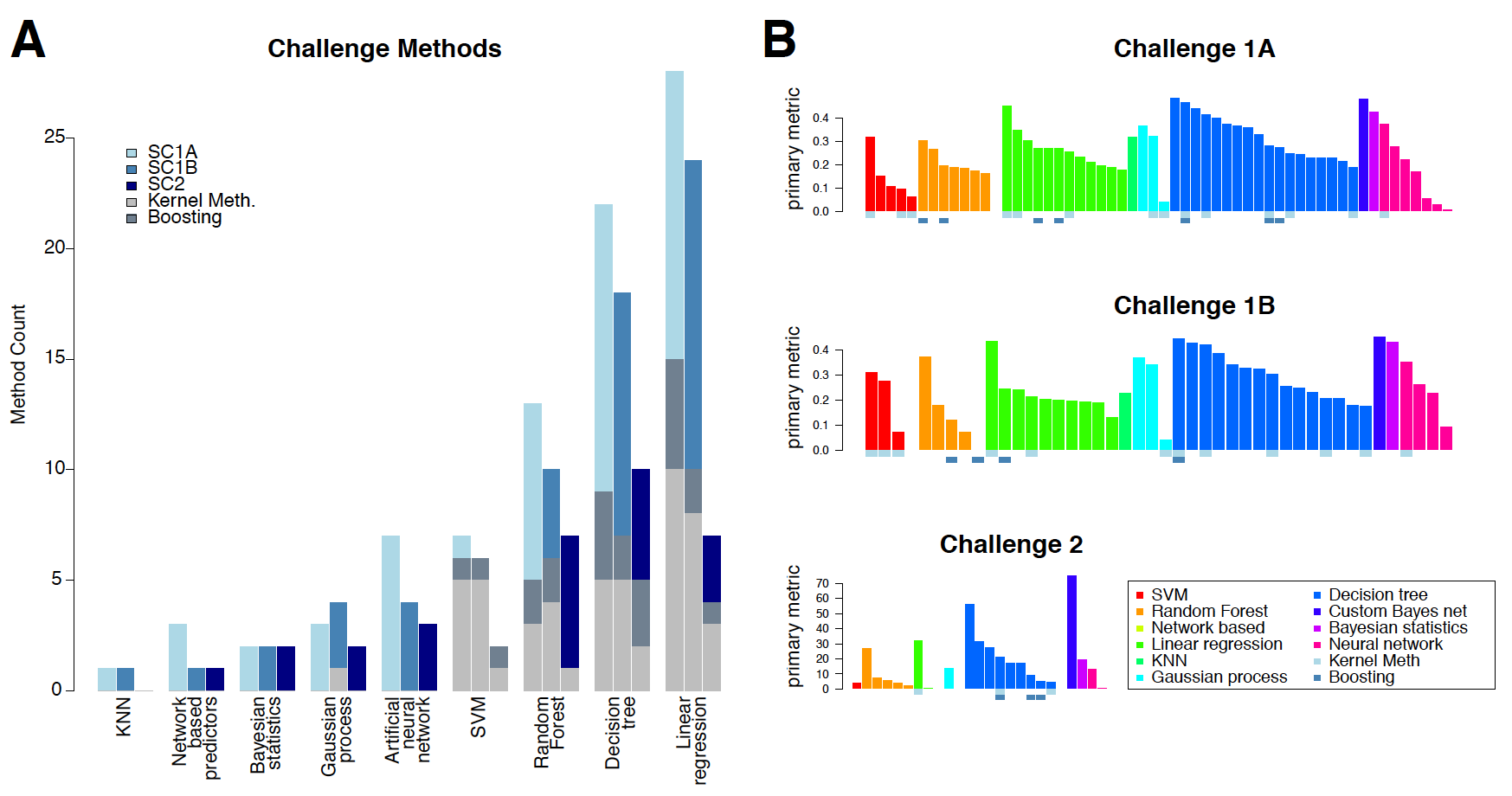


**Supplementary Figure 7**: (A) Methods used by participants in each sub-challenge. Each bar represents occurrence of the method in SC1A, SC1B, and SC2. (B) Performance of individual teams coloured by the primary type of machine learning method as reported by participants. Indicators below each bar show cases where kernel and boosting techniques were used with the primary method.


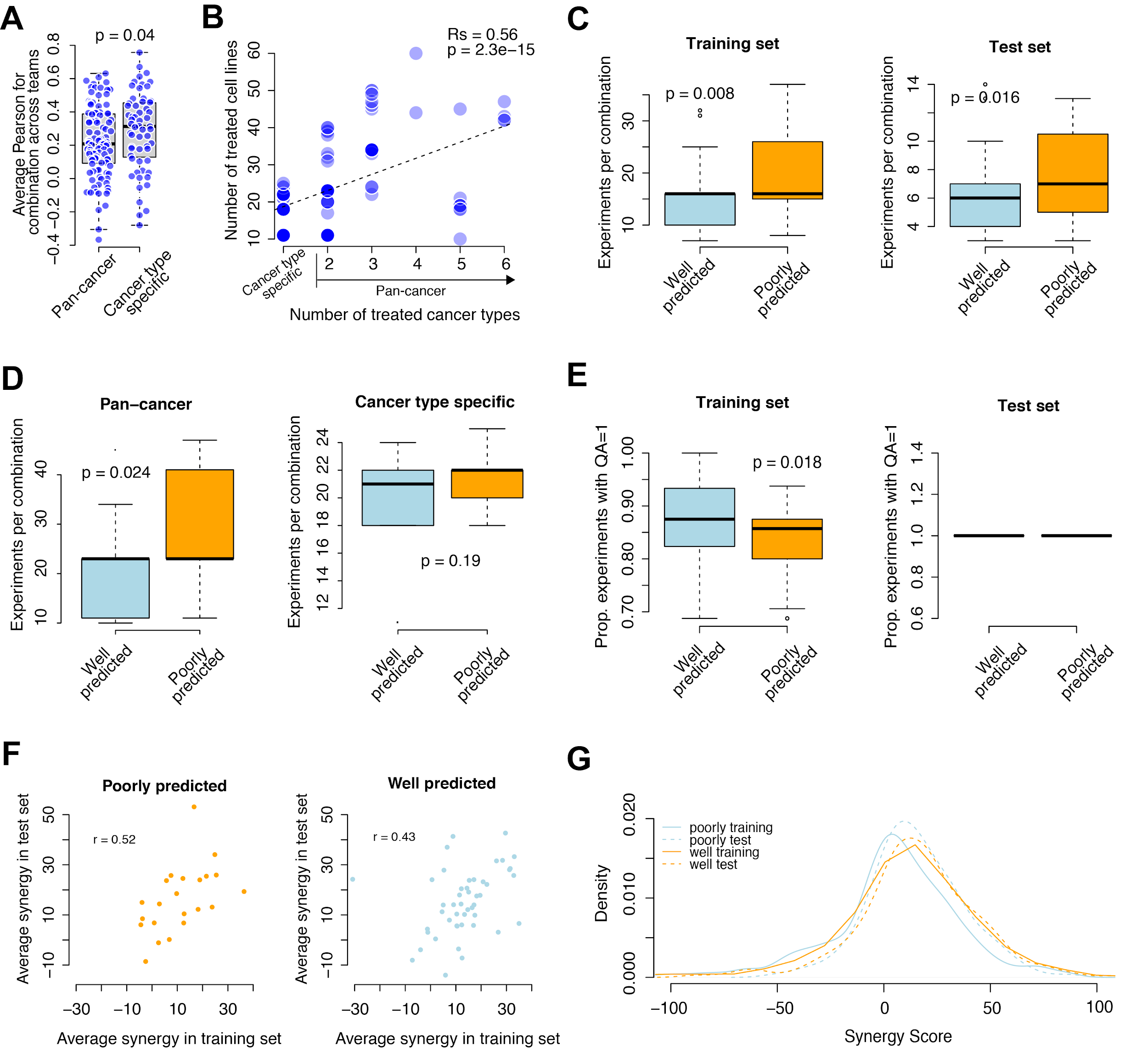


**Supplementary Figure 8**: (A) Average performances of drug combinations tested in cancer type specific setting vs pan-cancer. (B) Relationship between number treated cell lines and number of treated cancer types. Differences (t-test) between well and poorly predicted combinations based on: (C) number of experiments for each combination; (D) influence of cancer-type-specific vs pan-cancer prediction on training vs test performance; (E) proportion of experiments with high quality (QA=1); (F) comparison of average synergy score between training and test set for each of the well and poorly predicted combinations; and (G) distribution of synergy scores across combinations.


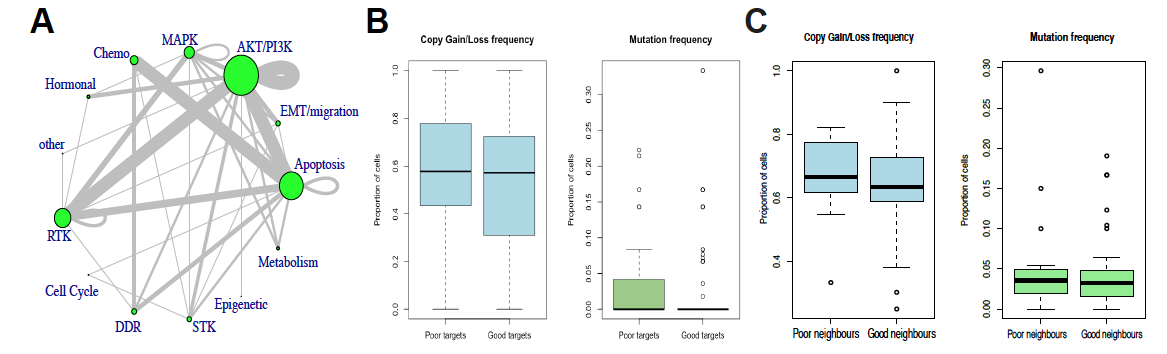


**Supplementary Figure 9**: (A) Combinations of all pathways targeted. Size of node is proportional to number of drugs targeting a specific pathway and width of edges is proportional to the number of drug combinations. (B) Copy number and somatic mutation frequency in the gene targets of drug combinations. (C) Copy number and somatic mutation frequency in the genes that are nearest interacting neighbors of the targets, as determined by *OmniPath*.


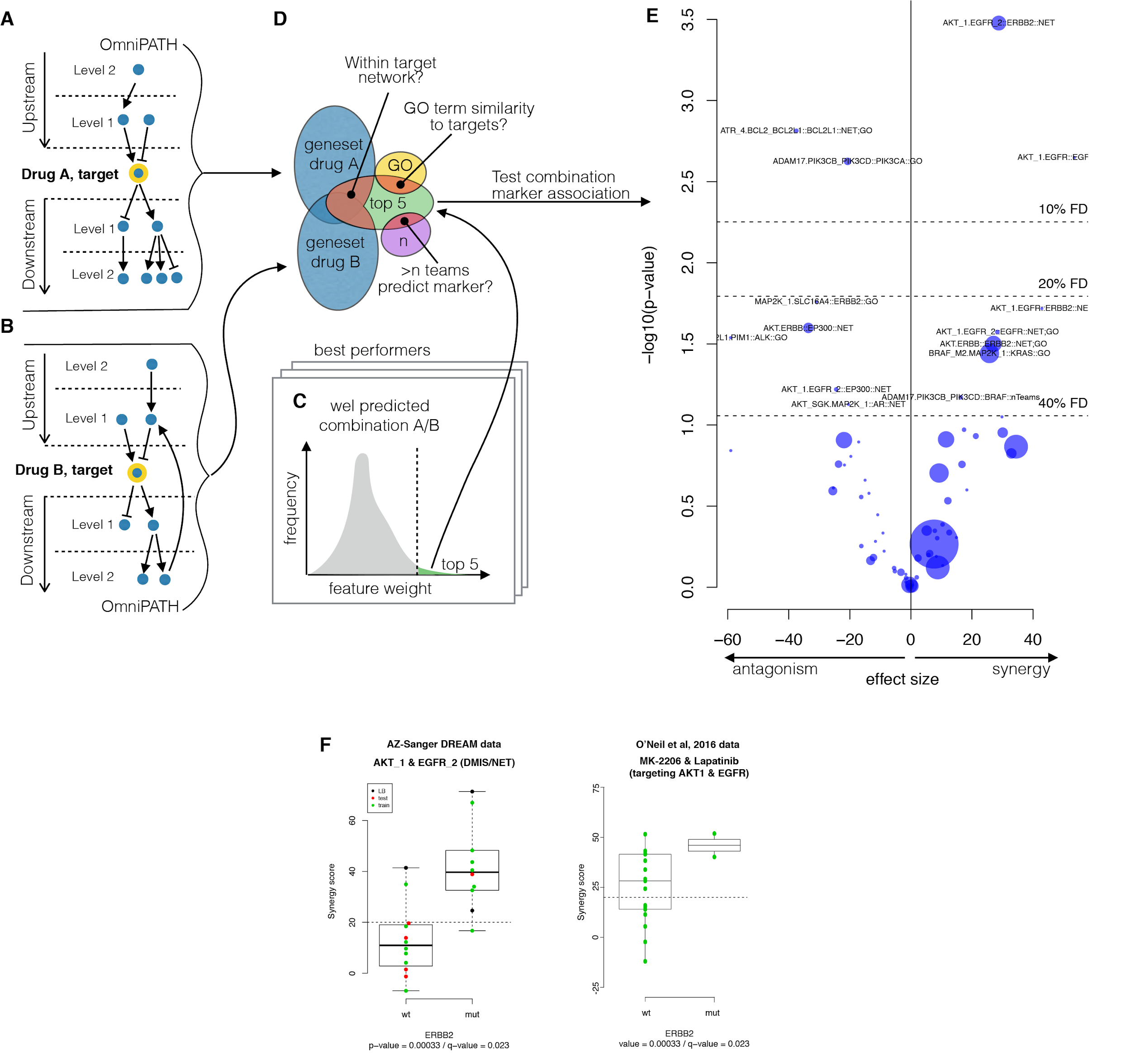


**Supplementary Figure 10**: Post-hoc analysis of synergy biomarkers. (A) and (B) shows the target centric exploration of 2 levels up- and downstream of the putative drug targets from each combination. (C) The top 5 ranked features from well predicted models were chosen for exploring their target enrichment. (D) Additionally, the top 5 features were further investigated if they had GO term similarity to the putative target larger than 0.7, or two independent teams relied on the same features. (E) This putative gene-to-combination association set was tested with an ANOVA model. (F) ERBB2 mutant cell lines showed synergy when treated with an EGFR and AKT inhibitor, which was independently validated with O’Neil et al. 2016.


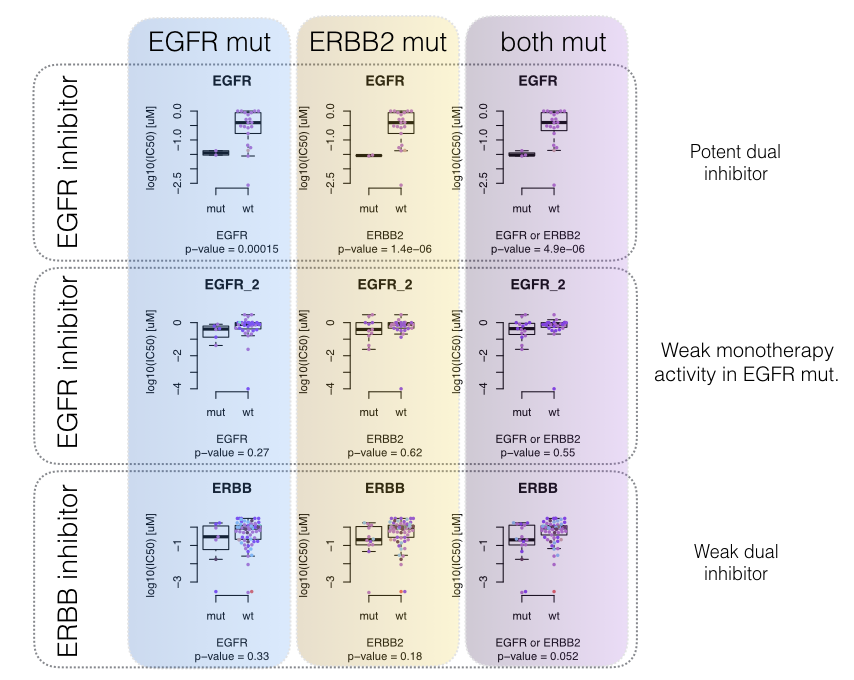


**Supplementary Figure 11**: Monotherapy markers of the inhibitors EGFR, EGFR_2 and ERBB. Exploration of EGFR, ERBB2 mutations and copy number changes alone and in combinations. Dot color represent different cancer types in our screen, while notably there is no enrichment, therefore no cancer type specific effect on this association.


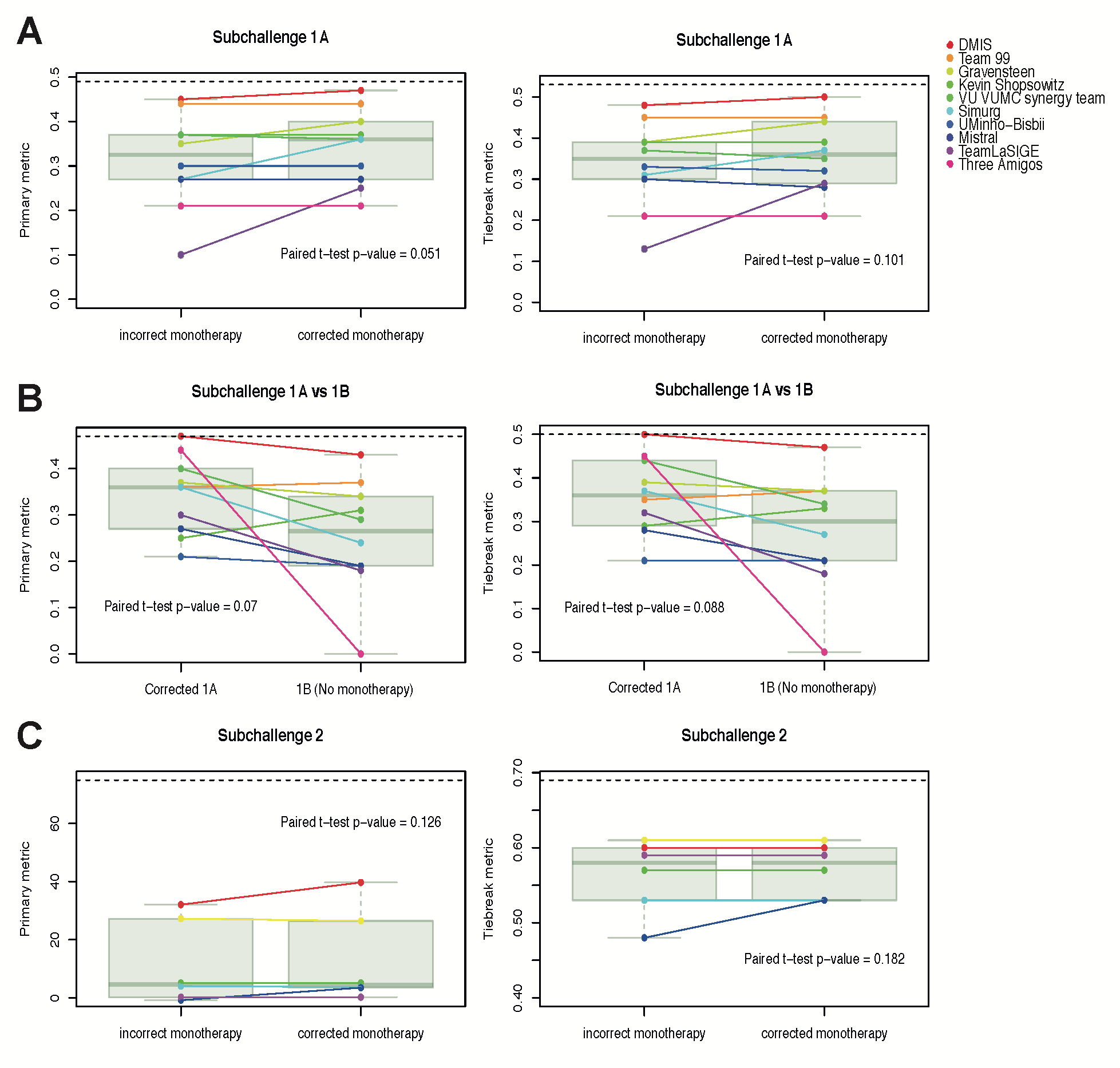


**Supplementary Figure 12**: Value of monotherapy on prediction performance. (A) Prediction performance of teams (dots) in SC1A when given correct and incorrect monotherapy data. (B) Comparison of performance between SC1A and SC1B for teams that had correct monotherapy data but could use it in SC1A and could not in SC1B. (C) Prediction performance of teams in SC2 when given correct and incorrect monotherapy data. Horizontal dashed line indicates the level of the top performing team.

Supplementary tables

**Supplementary Table 1: Overview of large-scale combination screening experiments**

|  | **DREAM - AstraZeneca** | **O’Neil et al.** | **NCI-ALMANAC (Holbeck et al.)** |
| --- | --- | --- | --- |
| **Compounds** | 119 | 38 | 104 |
| **Combinations** | 910 | 583 | 5,354 |
| **Cell lines** | 137* | 39 | 59 |
| **Drug concentrations tested** | 6 X 6 | 4 X 4 | 3 X 3 |
| **Day of measured response** | 5 | 4 | 2 |
| **Method for counting cells** | Sytox Green staining | Cell Titer-Glo | CellTiter-Glo |
| **Number of biological replicates** | 315 | 367 | 0 |
| **Compound overlap with DREAM** | - | 13 | 19 |
| **Combination overlap with DREAM** | - | 0 | 10 |
| **Combination-cell line overlap with DREAM** | - | 0 | 10 |

*** All cell lines released. 85 cell lines had complete genomic data**

**Supplementary Table 2: Prediction performance on O’Neil et al’s combinations screen**

|  |  | Performance: Average weighted Pearson Correlation | | | |
| --- | --- | --- | --- | --- | --- |
| Team | Method | All experiments | Same Cells | Similar Drug | Similar Combination |
| Mikhail | 1A model | 0.04 | 0.06 | 0.05 | 0.1 |
| Mikhail | 1B model | -0.05 | -0.07 | -0.02 | 0 |
| NorthAtlanticDream | 1A model | 0.05 | 0.05 | 0.05 | 0.03 |
| NorthAtlanticDream | 1B model | 0.03 | 0.07 | 0.05 | 0.07 |
| DMIS | new 1A model (Deep Learning) | 0.11 | 0.13 | 0.11 | 0.1 |
| DMIS | 1A model | 0.08 | 0.12 | 0.08 | 0.03 |
| DMIS | 1B model | -0.03 | 0.01 | 0 | -0.05 |
| Ensemble | Average 1A model | 0.13 | 0.17 | 0.13 | 0.11 |

## Supplementary Notes

1. Winning method (code freeze)
2. Scoring code is available online at <https://www.synapse.org/#!Synapse:syn4991619>

##

## Supplementary references

1. [O’Neil, J. *et al.* An Unbiased Oncology Compound Screen to Identify Novel Combination Strategies. *Mol. Cancer Ther.* **15**, 1155–1162 (2016).](http://paperpile.com/b/hsaAvY/MlDer)

2. [Breiman, L. Random Forests. *Mach. Learn.* **45**, 5–32 (2001).](http://paperpile.com/b/hsaAvY/obeGw)

3. [Guan, Y. *et al.* A genomewide functional network for the laboratory mouse. *PLoS Comput. Biol.* **4**, e1000165 (2008).](http://paperpile.com/b/hsaAvY/NzTQ2)

4. [Chen, T. & Guestrin, C. XGBoost: A Scalable Tree Boosting System. in *Proceedings of the 22Nd ACM SIGKDD International Conference on Knowledge Discovery and Data Mining* 785–794 (ACM, 2016).](http://paperpile.com/b/hsaAvY/oXd3k)

5. [Yadav, B., Wennerberg, K., Aittokallio, T. & Tang, J. Searching for Drug Synergy in Complex Dose–Response Landscapes Using an Interaction Potency Model. *Comput. Struct. Biotechnol. J.* **13**, 504–513 (2015).](http://paperpile.com/b/hsaAvY/OnM8x)

6. [Gene Ontology Consortium. Gene Ontology Consortium: going forward. *Nucleic Acids Res.* **43**, D1049–56 (2015).](http://paperpile.com/b/hsaAvY/w9zeh)

7. [Kanehisa, M., Sato, Y., Kawashima, M., Furumichi, M. & Tanabe, M. KEGG as a reference resource for gene and protein annotation. *Nucleic Acids Res.* **44**, D457–62 (2016).](http://paperpile.com/b/hsaAvY/KqWNw)

8. [Babur, Ö. *et al.* Systematic identification of cancer driving signaling pathways based on mutual exclusivity of genomic alterations. *Genome Biol.* **16**, 45 (2015).](http://paperpile.com/b/hsaAvY/wzE3N)

9. [Sun, Y. *et al.* Combining genomic and network characteristics for extended capability in predicting synergistic drugs for cancer. *Nat. Commun.* **6**, 8481 (2015).](http://paperpile.com/b/hsaAvY/IQMKT)

10. [Woods, D. & Turchi, J. J. Chemotherapy induced DNA damage response: convergence of drugs and pathways. *Cancer Biol. Ther.* **14**, 379–389 (2013).](http://paperpile.com/b/hsaAvY/EY1CY)

11. [An, O., Dall’Olio, G. M., Mourikis, T. P. & Ciccarelli, F. D. NCG 5.0: updates of a manually curated repository of cancer genes and associated properties from cancer mutational screenings. *Nucleic Acids Res.* **44**, D992–9 (2016).](http://paperpile.com/b/hsaAvY/FsIHH)

12. [Wagner, A. H. *et al.* DGIdb 2.0: mining clinically relevant drug–gene interactions. *Nucleic Acids Res.* **44**, D1036–D1044 (2016).](http://paperpile.com/b/hsaAvY/NXJwB)

13. [Yang, W. *et al.* Genomics of Drug Sensitivity in Cancer (GDSC): a resource for therapeutic biomarker discovery in cancer cells. *Nucleic Acids Res.* **41**, D955–61 (2013).](http://paperpile.com/b/hsaAvY/qXKmo)

14. [Caruana, R., Niculescu-Mizil, A., Crew, G. & Ksikes, A. Ensemble Selection from Libraries of Models. in *Proceedings of the Twenty-first International Conference on Machine Learning* 18– (ACM, 2004).](http://paperpile.com/b/hsaAvY/Os15s)

15. [Lee, S. *et al.* BEST: Next-Generation Biomedical Entity Search Tool for Knowledge Discovery from Biomedical Literature. *PLoS One* **11**, e0164680 (2016).](http://paperpile.com/b/hsaAvY/WYk8C)

16. [Mikolov, T., Sutskever, I., Chen, K., Corrado, G. S. & Dean, J. Distributed Representations of Words and Phrases and their Compositionality. in *Advances in Neural Information Processing Systems 26* (eds. Burges, C. J. C., Bottou, L., Welling, M., Ghahramani, Z. & Weinberger, K. Q.) 3111–3119 (Curran Associates, Inc., 2013).](http://paperpile.com/b/hsaAvY/Z1k8z)

17. [Asgari, E. & Mofrad, M. R. K. Continuous Distributed Representation of Biological Sequences for Deep Proteomics and Genomics. *PLoS One* **10**, e0141287 (2015).](http://paperpile.com/b/hsaAvY/kjoJa)
