# Supplementary material for: Community assessment to advance computational prediction of cancer drug combinations in a pharmacogenomic screen
Source: Nat Commun. 2019 Jun 17;10:2674. doi: 10.1038/s41467-019-09799-2 (PMC6572829; doi:10.1038/s41467-019-09799-2)
Supplement: Supplementary file 2 — Description of Additional Supplementary Files [file 41467_2019_9799_MOESM2_ESM.docx]

**Description of Supplementary Files**

**File Name:** Supplementary Data 1.

**Description:** The processed pharmacology data.

**File Name:** Supplementary Data 2.

**Description:** The synergy biomarker survey.

**File Name:** Supplementary Data 3.

**Description:** The synergy biomarker post hoc analysis.
